# Expression-based discovery of candidate ovule development regulators through transcriptional profiling of ovule mutants

**DOI:** 10.1186/1471-2229-9-29

**Published:** 2009-03-16

**Authors:** Debra J Skinner, Charles S Gasser

**Affiliations:** 1 Department of Molecular and Cellular Biology, University of California, Davis, CA 95616, USA; 2 Department of Crop Science, University of Illinois, Urbana, IL 61801, USA

## Abstract

**Background:**

*Arabidopsis *ovules comprise four morphologically distinct parts: the nucellus, which contains the embryo sac, two integuments that become the seed coat, and the funiculus that anchors the ovule within the carpel. Analysis of developmental mutants has shown that ovule morphogenesis relies on tightly regulated genetic interactions that can serve as a model for developmental regulation. Redundancy, pleiotropic effects and subtle phenotypes may preclude identification of mutants affecting some processes in screens for phenotypic changes. Expression-based gene discovery can be used access such obscured genes.

**Results:**

Affymetrix microarrays were used for expression-based gene discovery to identify sets of genes expressed in either or both integuments. The genes were identified by comparison of pistil mRNA from wild type with mRNA from two mutants; *inner no outer *(*ino*, which lacks the outer integument), and *aintegumenta *(*ant*, which lacks both integuments). Pools of pistils representing early and late stages of ovule development were evaluated and data from the three genotypes were used to designate genes that were predominantly expressed in the integuments using pair-wise and cluster analyses. Approximately two hundred genes were found to have a high probability of preferential expression in these structures, and the predictive nature of the expression classes was confirmed with reverse transcriptase polymerase chain reaction and *in situ *hybridization.

**Conclusion:**

The results showed that it was possible to use a mutant, *ant*, with broad effects on plant phenotype to identify genes expressed specifically in ovules, when coupled with predictions from known gene expression patterns, or in combination with a more specific mutant, *ino*. Robust microarray averaging (RMA) analysis of array data provided the most reliable comparisons, especially for weakly expressed genes. The studies yielded an over-abundance of transcriptional regulators in the identified genes, and these form a set of candidate genes for evaluation of roles in ovule development using reverse genetics.

## Background

**Figure 1:**
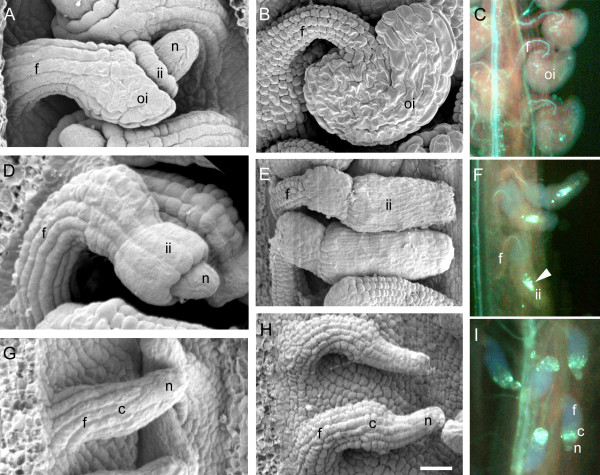
**Ovule phenotypes of wild type, *ino *and *ant***. A comparison of wild type (A – C) ovule development with *ino *(D – F) and *ant *(G – I) using scanning electron and fluorescence microscopy. Ovules are shown at developmental stage 2-IV (A, D, G), and 4-IV (B, E, H). (A, B) In wild type ovules, the two integuments grow as sheaths around the nucellus until it is fully enclosed and the outer integument envelopes the inner integument. (D, E) In contrast, *ino *mutant ovules show only inner integument growth and this structure encloses the nucellus but does not cause curvature of the ovule at maturity. (G, H) *ant *ovules do not initiate integuments but do elongate and form a swollen region at the chalaza. The *ant *nucellus is naked at maturity. (C, F, I) Ovules at anthesis were cleared and stained for callose accumulation to identify non-functional embryo sacs that can be seen as brightly fluorescing structures in *ino *mutants (arrow, F) and that are absent in wild type (C). Mature *ant *ovules (I) lack embryo sacs, but show callose fluorescence associated with chalaza and nucellus. c, chalaza; f, funiculus; ii, inner integument; oi, outer integument; n, nucellus. Scale bar = 15 μm in A, D, and G; 20 μm in H; 25 μm in B and E; and 45 μm in C, F, and I.

Ovules, the precursors to seeds, are an important focus of study to better understand plant development within a unique reproductive context. Ovules are highly specialized for reproductive function, but the typical angiosperm ovule, as found in Arabidopsis, is relatively simple morphologically. Development of the ovule within the carpel is well described, [1-5], beginning with primordia emergence from the marginal placentas of the carpels (floral stage 9, ovule stage 1). The primordia have three regions, the distal region or nucellus, marked by the formation of the large megaspore mother cell, the central or chalaza region indicated by the emergence of the two integuments, and the proximal region which forms the funiculus supporting the ovule (Figure 1A; floral stage 10, ovule early stage 2). The inner integument initiates as a ring from divisions in the L1, while the outer integument derives from divisions on the gynobasal side of the ovule below the inner integument. The integuments grow together to enclose the nucellus and when this has occurred the embryo sac develops from a meiotic product of the megasporocyte. The integuments continue to differentiate with the outer and inner integument cells changing in appearance in preparation for the integuments roles in pollen tube attraction [6] and formation of the seed coat.

Our knowledge of the genes involved in ovule development has benefited from three complementary approaches. Mutants with altered integument morphogenesis such as *bell1 *(*bel1*) and *inner no outer *(*ino*) were discovered in screens for sterility [1,7-10]. Systematic reverse genetics analysis of families of transcription factors has also yielded important ovule regulators, including the MADS domain proteins encoded by *SHATTERPROOF *(*SHP*)*1/2 *and *SEEDSTICK (STK) *[11,12]. Finally, several genes identified through their action in other organs or processes were subsequently shown to have important effects in ovules, including *WUSCHEL (WUS) *and *PHABULOSA (PHB) *[13,14]. Identification of such genes and analysis of their interactions have permitted the construction of models of ovule development, including specification of regional ovule identity, integument identity and outgrowth, and asymmetric growth of the outer integument, reviewed in [15].

The two integuments are particularly interesting as a focus of study as their evolutionary origins are unclear and are likely to be separate, the inner integument from sterile branches or telomes, and the outer integument from lateral structures similar to leaves [16,17]. Despite the recent advances, control of several aspects of ovule development, such as inner integument patterning and integument morphogenesis, remains poorly understood. Further mutant screens to uncover regulatory genes may have limited success as some phenotypes may not cause sterility, and pleiotropic effects that lead to loss of flowers would obscure ovule effects. A further problem results from redundancy between gene families or pathways, which has been shown for diverse Arabidopsis developmental regulators such as the *SEPALLATA (SEP)*/*AGAMOUS (AG) *clade of MADS domain genes [11,18], the *NO APICAL MERISTEM (NAM) *family genes, *CUP-SHAPED COTYLEDONS (CUC)1 *and *2 *[19], and the *KANADI (KAN) *genes [20]. An alternative to such forward genetic approaches is the expression-based discovery of integument-expressed genes. Research on the genes described above has shown that developmental regulators often have specific and restricted spatial and temporal domains of expression and this concept has been exploited in strategies to find such genes using subtractive hybridization, differential display, cDNA and oligonucleotide microarrays, and technologies such as Serial Analysis of Gene Expression (SAGE) and Massively Parallel Signature Sequencing (MPSS) [21-25].

Microarrays have been successfully used to identify genes expressed in specific structures. Some studies have utilized isolated cell types or organs, such as guard cells or pollen for this purpose [26-28]. In other studies, developmental mutants that have homeotic changes or loss of structures have been used through comparisons to wild type to identify genes expressed in those structures [29-34]. Two ovule mutants have phenotypic properties that would enable a microarray approach to integument gene identification. The *ino-1 *mutant has an almost complete loss of the outer integument [7]. The *INO *gene encodes a YABBY domain protein important in polarity and growth of the outer integument [35]. The *AINTEGUMENTA *(*ANT*) gene is known to be expressed in and required for proper outgrowth of organ primordia and, in particular, *ant *mutants fail to initiate integument primordia [36-39]. Because these mutants lack integuments, any gene that is expressed mostly in these structures should be at a much lower abundance relative to wild type in the set of mRNAs isolated from these mutants. This approach would not be expected to identify only genes that are direct targets of *INO *and *ANT*, but rather a set of genes downstream of these that are expressed in the structures that are absent in the mutants.

We used microarrays to evaluate differences in gene expression between wild-type carpels and those of *ino-1 *and *ant-4*. Approximately nine hundred genes were identified that were predicted to be expressed in placenta or ovules, with two hundred twenty-two of these genes reliably predicted to be in the ovule primordia or integuments based on high fold changes or support from both mutants. Among these are genes that are known to have integument-specific activity, demonstrating that the approach can detect genes important for ovule function. The results were validated through quantitative polymerase chain reaction and *in situ *hybridization for a subset of the genes. These results will help build a more detailed picture of the processes involved in integument morphogenesis, and, through further research on candidate genes, will yield a greater understanding of the mechanisms of regulation of ovule morphogenesis.

## Results

### Mutants used for comparative expression profiling

The *ino *and *ant *mutants were chosen for array analysis due to their ovule phenotypes. Ovules of strong *ino *mutants have only an inner integument, and do not curve as in wildtype (Figure 1D, E) [7,35]. The general role of the *ANT *gene in all above ground organs is to promote the formation and growth of primordia, and to regulate that growth to control the size of plant organs [36-41]. For most organs these functions are partially redundant with other genes, but in *ant *mutants the integument primordia fail to initiate, and mature ovules have only a slightly enlarged chalaza between the funiculus and nucellus (Figure 1G, H).

In addition to the integument phenotypes, both mutants are affected in formation of mature embryo sacs, leading to partial and complete sterility for *ino *and *ant *respectively. The viability of mature embryo sacs was estimated using decolorized aniline blue staining for callose, which accumulates in defective embryo sacs [42,43] (Figure 1C, F, I). 13% of *ino *mutant embryo sacs (n = 100) did not show an accumulation of callose staining and approximately 5 seeds per silique (compared with 45–50 for wild type) were formed under experimental growth conditions indicating that only approximately one in ten embryos sacs were functional. This is in agreement with microscopic analysis indicating that structurally normal embryo sacs could be formed in *ino *mutants [7]. For the *ant-4 *allele, there are fewer ovules per carpel than in wild type, and sporogenesis was not observed to proceed beyond the megaspore mother cell stage [7] resulting in complete female sterility [36,37]. In addition, pistil size and stigma cell number were reduced, and carpels could be partially unfused [44,45].

### Expression Profiling

The pistil expression profiles of *ino *and *ant *were compared with each other and to wild type (Landsberg *erecta*, Ler), with the following predicted gene expression profiles. As the outer integument is missing in both mutants, genes that are preferentially expressed there should exhibit absent or significantly decreased expression in both *ino *and *ant *samples, relative to wild type. In contrast, an inner integument-expressed gene should exhibit absent or decreased expression in *ant *but would be unchanged in *ino *samples as the inner integument is still present. A gene that is expressed in both integuments is likely to show reduced expression in both mutants, with a greater reduction in *ant *samples as these lack both integuments. However, gene expression changes may be also caused by defective embryo sac formation in *ino *and *ant *and by the reduction in ovule number and effects on carpels in *ant*. By noting the expression level changes of genes that are known to be expressed in these areas it may be possible to define a pattern that identifies such genes for exclusion.

**Figure 2:**
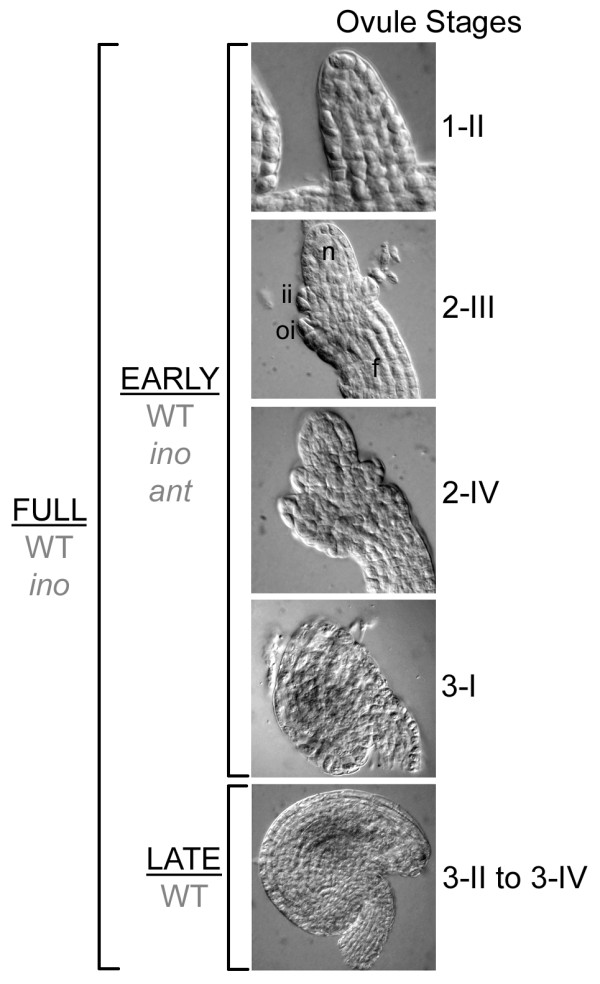
**Stages of ovule development collected in the pistil pools**. Differential interference contrast images of wild-type ovules representing stages included in the pools. The FULL pools of pistils contained ovules from stage 1-II, through stage 3-IV ("maturity"). The EARLY pools of pistils contained ovules from stage 1-II through stage 3-I, when the integuments just cover the nucellus. The LATE pool included ovule stages 3-II to 3-IV during which here is little change in ovule shape. The genotypes that were collected for each pool are indicated in grey. Ovules stages are based on Schneitz et al. [2]. f, funiculus; ii, inner integument; oi, outer integument; n, nucellus.

Three different developmental classes of pooled pistils were used. The FULL (F) pool, collected from wild type (WT) and *ino*, contained pistils from the stage at which ovule primordia are emerging (floral stage 9, ovule stage 1-II), up to mature ovules, just prior to anthesis (Figure 2; floral stage 12, ovule stage 3-IV). Samples containing fewer stages were collected to decrease the complexity of the samples to provide better resolution of expression differences and to evaluate the temporal expression patterns of selected genes. The EARLY (E) pool, collected from all three genotypes, included the youngest stages described above up to the point when the integuments first enclose the nucellus (floral stage 11, ovule stage 3-I), while the LATE (L) pool, collected from WT, captured the remaining stages after the integuments enclose the nucellus up to anthesis. Three biological replicates of each sample were used, with the exception of the WT L arrays, which had two replicates.

Affymetrix ATH1 Genome Arrays representing approximately 23,000 genes [46,47] were hybridized with the WT, *ino *and *ant *samples. The data from these arrays were processed using Robust Multiarray Averaging (RMA) [48], as well as with dchip and Microarray Suite 5.0 (MAS) in order to determine which method would be most appropriate for the data analysis. Scatterplots between replicates (Additional file 1) and correlation coefficients (Additional file 2) showed that the replicates were very similar to each other (r = 0.9930 - 0.9979 for RMA) and that RMA produced the smallest variance between replicates, particularly at low levels of expression. Comparisons between genotypes were made with the RMA processed data using a moderated t-test in the *limma *program (*affylmGUI*) [49-52], which stabilizes variances when few replicates are used, and has been used to analyze data in other plant microarray experiments with similar numbers of replicates [34,53-55]. A multiple testing adjustment of p-values was obtained by conversion to q-values, where a confidence level of 0.01 was used, giving a false discovery rate of 1% [56]. The dchip processed data were also used for genotype comparisons using the modified fold change method, where the fold change threshold was 1.2, using the lower bound of the 90% confidence interval for fold change. Using the two statistical tests, there were more genes identified as significantly changed between *ant *E and wildtype with the RMA-*limma *test than with the dchip test (3537 and 1672 respectively) and greater than 50% of these genes were uniquely identified by a single method (Additional file 3). The *limma *test with RMA processed data was successful at identifying seventeen genes known to be expressed in ovules while the dchip fold change method was not as successful (five of seventeen genes failed to be identified) (Additional file 4). This indicated that the RMA-*limma *method was more appropriate for the task of identifying ovule-expressed genes than the dchip method. Based on these results, RMA processed data were used for further expression analysis described below.

Pairwise comparisons between the mutant and the baseline wild type arrays (WT F and *ino *F, WT E and *ino *E, and WT E and *ant *E) showed that many more genes were changed in the *ant *E samples (up to 14 times the number identified with *ino *E) (Table 1), which was predictable from the more perturbed phenotype of *ant *mutants and the wider spectrum of action of the *ANT *gene. In addition, there were more genes identified in the *ino *F comparison than in the *ino *E comparison, which indicates that there are several identified genes expressed in later stages of ovule development. Finally, there were at least as many genes with increased expression in the EARLY mutant arrays as there were with decreased expression. This is in contrast to the *ino *F comparison, where more genes exhibited decreased expression in the mutant.

**Table 1:** Number of genes significantly changed between mutants and wildtype

**Pairwise tests**	**# of genes**	**Subcategory**^a^	**# of genes**
WT F vs *ino *F	**474**	Up	158
		Down	316
WT E vs *ino *E	**243**	Up	120
		Down	123
WT E vs *ant *E	**3537**	Up	1820
		Down	1717

The total number of significantly changed genes between the indicated genotypes are presented. These were then divided into those genes where expression increased in the mutant (up), or that showed lower expression in the mutant (down). The "down" genes are the desired genes.

Based on the EARLY array hybridizations, expression of 1717 genes was significantly decreased in the *ant *mutant relative to WT, and these genes formed a set from which putative inner and outer integument genes were identified based on their levels of expression in *ino*. From this set, eight hundred putative inner integument genes were identified based on their showing a significant decrease in *ant *E samples relative to *ino *E, but no difference between WT E and *ino *E (WT = *ino *> *ant*). Of the 1717 genes reduced in *ant*, eighty-nine genes showed a decrease in *ino *E relative to WT and these were further divided into a putative outer integument set of fifty-eight genes that showed no difference between *ino *E and *ant *E (WT > *ino *= *ant*) and a set of twenty-five genes that showed a further significant decrease in *ant *E and are therefore likely to have expression in both integuments (WT > *ino *> *ant*). These groups of genes were clustered with Kohonen self-organizing maps as implemented in GeneCluster 2.1.7 (SOM) [57], and Broad Institute  using all the arrays (Additional file 5). Observing gene levels in the *ino *F arrays allowed for extrapolation of the *ino *E inferences and use of the LATE arrays showed whether expression of a particular gene is maintained later in development. Genes with known expression were used to understand the nature of the observed expression patterns and to set thresholds that reflect specific expression patterns, as described below.

### Genes putatively express in the inner integument

The set of genes our analysis indicated were expressed in the inner integument included several genes previously shown to be expressed in ovule primordia or integuments. These included *PHB*, an inner integument-expressed gene, indicating that the comparisons could identify desired genes. There was minor overlap with putative gametophyte expressed genes (83 of 1278) identified using mature *nozzle/sporocyteless *(*nzz/spl*) mutant ovules [33,58,59] and *coatlique *mutant gynoecia [34]. However, some genes expressed in specific cells of the embryo sac, such as *WUSHCEL-RELATED HOMEOBOX *2 (*WOX2*) and *WOX8 *[60] or during meiosis (seven genes including homologs of *SPO11 *and *RAD51*) [61,62], show no differential expression in our *ant*-hybridized arrays. A few genes were previously identified as expressed in stigmatic papillae and transmitting tract using arrays (seven of 140 identified) [31]. Therefore, most of the differentiation that occurs in the stigma, transmitting tract and gametophyte was not captured by this experiment, leaving the loss of the integuments, reduction in ovule number and reduced growth of the medial regions as likely causes of the identification of the large number of genes with small changes in *ant*.

The interpretation of the cluster profiles for the 800 putative integument genes depended on whether the mutant expression was considered absent (which indicated the specificity of expression), the inclusion of known indicator genes for each cluster (Additional file 6) and evaluation of expression levels in *ino*. At least six genes fall to very low levels in *ant*, and therefore could be integument specific, while two thirds of the genes appear to be expressed more highly during early stages of pistil development since the WT E level is higher than the WT L level.

**Figure 3:**
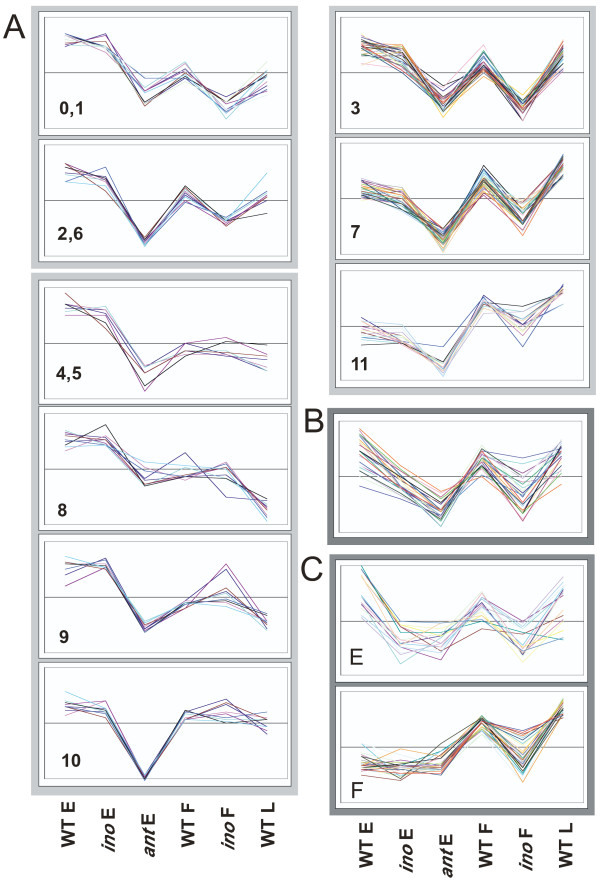
**Groups of inner and outer integument expressed genes identified by SOM clustering and significant differences in expression**. The expression profiles of genes that showed significant changes and were more than two-fold changed in the mutants are shown grouped by predicted location of expression and cluster. The mean values for each gene were standardized to a mean of 0 and standard deviation of 1 (z-transformation), in order to focus on expression changes and not magnitude of expression. SOM cluster numbers (Additional file 5) are indicated at the bottom left of the graphs where applicable. (A) Genes likely to be expressed in the inner integument or other regions affected by the *ant *mutant are separated into 3 groups with different patterns. (B) Genes likely to be expressed in both integuments show a steady decrease in expression from WT E through *ino *E to *ant *E. (C) Genes likely to be expressed in the outer integument or at late stages in the embryo sac. The EARLY ("E") group is defined by lower expression levels in *ino *E, with similar levels in *ant *E while the FULL ("F") group contains those genes that only showed significant differences between WT F and *ino *F, and not at the early stages.

SOM clusters 4, 5 and 8 – 10 (Figure 3A) contain a total of 310 genes that show little or no change between WT and *ino *arrays even at later stages and that have higher WT E than WT L levels (except cluster 10), indicating little or no outer integument or embryo sac expression later in development, and more expression early in development. Known genes in this group are expressed in medial regions, placenta and ovule primordia, for example *CUC2, PERIANTHIA *(*PAN*) and *NUBBIN *[19,63,64], while others also show some expression in integument primordia, such as *BEL1*, *SPATULA *(*SPT*) and *PIN-FORMED 1 *(*PIN1*) [10,65,66]. For these genes, outer integument expression is too low to be discerned in the *ino *arrays relative to the overall expression levels in the pistil. The patterns can be roughly separated on the basis of fold change: expression in ovule primordia regions results in smaller changes (approximately -1.4) and ovule and integument expression results in slightly higher fold changes.

The remaining clusters all show changes in *ino *E arrays which were not considered statistically significant but do show a consistent pattern, and are split into two groups by their WT L and *ino *F expression levels, which are significantly lower than WT F levels in some cases. For clusters 0, 1, 2 and 6 the WT L expression is less than the WT E level (Figure 3A) indicating that gene expression does not rise or expand in later stages and the decrease in *ino *levels may indicate some expression in the outer integument. Accordingly, this group contains genes such as *AINTEGUMENTA-LIKE 5 *(*AIL5*) and *ERECTA-LIKE 2 *(*ERL2*), expressed in placenta, ovule and integument primordia [67,68] and L1 specific genes such as *PROTODERMAL FACTOR 2 *(*PDF2*) and *MERISTEM LAYER 1 *(*ATML1*) expressed in ovule primordia and the L1-derived integuments [69,70]. In addition, twenty seven embryo sac genes identified either by Yu *et al *[33] or by Johnston *et al *[34] were found in this set of genes, which could also be a cause of decreased *ino *E levels. Cluster 2 also contains *PHB*, previously shown to be downregulated in *ant *gynoecia [40], whose slight decrease in expression in the *ino *arrays could be reflecting post-transcriptional regulation of the mRNA in the outer integument [14,71].

The third group, clusters 3, 7 and 11, in which expression seems to increase towards later stages of pistil development, also shows decreases in the *ino *F arrays, with larger decreases in clusters 3 and 7 (Figure 3A). Such genes are likely to have early carpel or ovule expression as well as later outer integument expression, and remain expressed in later stages, as is seen with the cluster 3 gene, *FIDDLEHEAD *(*FDH*) [72], and cluster 11 genes *ERL1*, which is expressed throughout early carpels and later resolves to expression in the ovules [68], and *PRETTY FEW SEEDS *(*PFS2*), expressed in carpel and ovule primordia, and in the chalaza, integument primordia and nucellus [73]. These genes have more ovule specific expression and also show higher fold changes, which is likely to be correlated.

In summary, the known genes in this set of 800 genes encompass a wide variety of expression patterns, whose common thread seems to be expression in ovule primordia. On the basis of the clustering results, genes expressed primarily in the inner integument would be expected to have patterns similar those genes in clusters 4, 5, 8, 9, and 10. These groups are also likely to be populated with genes that have expression in placenta and ovule primordia. Any decrease in *ino *arrays appears to signify a wider expression pattern that includes ovule and integument primordia, and expression that is maintained later in development likely shows that the expression is not limited to primordial cells. With a few exceptions, more specific or prolonged ovule expression leads to higher fold changes between wild type and *ant*.

### Genes putatively expressed in the outer integument

The fifty-eight genes that are decreased in both *ino *and *ant *to a similar level (Figure 3C) are considered good candidates for expression in the outer integument, and accordingly, the *APETALA 3 *(*AP3*) gene, known to be expressed in the outer integument, was identified [74]. Similar to the genes described above, lower fold changes could imply general carpel expression combined with elevated or more specific outer integument expression, as seen with the *SHP2 *gene, also found in this group. *SHP2 *acts with related genes to specify ovule development, and is also expressed early in carpel development [11,75,76]. Mutations in *RABBIT EARS *(*RBE*), produce a phenotype in which the growth of the outer integument is aberrant, with the inner integument being affected at later stages [77,78]. Expression of this gene has been described as being in both integuments [78], but this is not reflected by the measurements on the arrays, which show similar decreases in both *ino *and *ant *relative to wild type. There are at least three other uncharacterized transcription factor genes that would be good candidates for activity in the outer integument. These encode an AP2 domain protein, a ZF-HD protein and a myb domain protein.

Putative outer integument genes are also contributed by a comparison of the WT F and *ino F *arrays, with one hundred forty three genes identified only by these arrays. These are good candidates for being expressed in the later stages of outer integument development, and may be involved in differentiation of the cell layers in preparation for pollen reception or seed coat development. However, genes that are predominantly and strongly expressed in the embryo sac at late stages will share this pattern, as evidenced by the overlap (50 genes, 35%) with putative gametophyte expressed genes [33,34]. Therefore all the identified genes will require further validation of expression pattern. A total of seventeen putative outer integument genes dropped to very low levels in *ino *indicating specific expression in the outer integument.

### Genes putatively expressed in both integuments

Twenty-five genes exhibited expression patterns expected for expression in both integuments, with *ino *expression lower than wild type and *ant *lower than *ino *(Figure 3B). Most such genes were greater than two-fold changed from wild type to *ant*, and all showed a reduction in *ino *F as well as in *ino *E, with variation in WT L levels. As expected, this group contains the *INO *gene, which is expressed briefly in the *ino *mutant [79]. Also detected here is the *SUPERMAN *(*SUP*) gene, involved in regulation of *INO *[79,80] and integument growth, although expression in integuments has not been observed [81-83]. For At4g12960, only inner integument expression has been described, at late stages [30], but the array measurements predict expression in both the inner and outer integuments at earlier stages. For this gene and *SUP *it is possible that the loss of the outer integument affects gene expression in the inner integument.

### Summary of analysis

While all the above genes are candidates for expression in the integuments, only those genes with significant expression changes from wild type in both *ino *and *ant*, or those with a 2-fold change level from wild type were examined closely (207 genes). This selection was made based on the observation that known expression patterns that were most specific to ovules had higher fold changes. This leaves 132 putative inner integument genes (Additional file 7), retaining known ovule expressed genes such as *BEL1, PFS2, ETTIN *(*ETT*), *PAN *and *AIL5*, but excluding genes with wider carpel expression (such as *PHB *and *CUC2*), L1 expressed genes, and other placenta and primordia genes such as *FDH, MONOPTEROS, ATCEL2 *and *SPT*. The outer integument group is reduced to 50 genes, removing genes such as *SHP2 *(FC = -1.3) whose expression is not specific to the outer integument, but retaining the *AP3 *and *RBE *genes (Additional file 8). The 25 genes that show decreases in both *ino *and *ant *are all retained and listed in Additional file 9. The expression profiles of these genes from the arrays are shown in Figure 3, grouped by general expression changes into 'outer integument', 'both integuments' and 'inner integument and primordia expression' groups, and then into subgroups based on analysis information above.

### Functional categorization of the discovered genes

**Figure 4:**
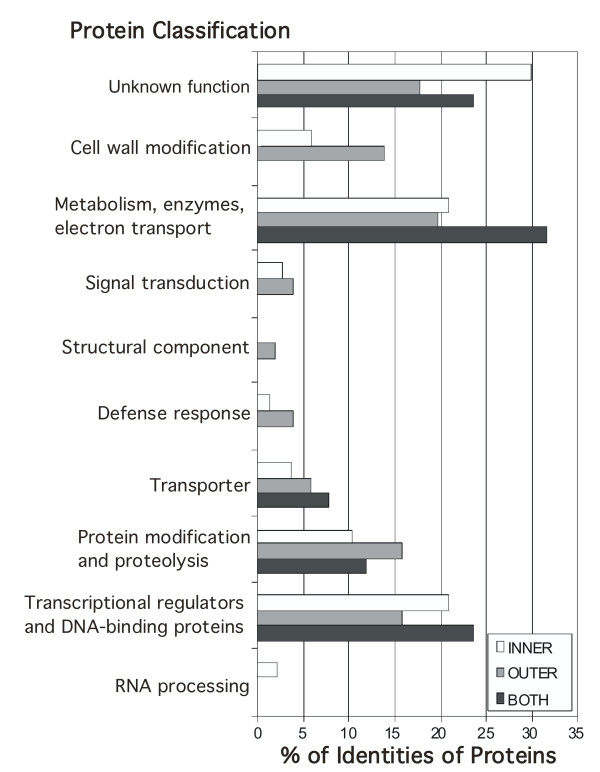
**Classification of identified genes by protein type and function**. Proteins were classified into categories using GO annotations and published information and the percentages of each category encoded by the genes in each integument group are shown. 'Unknown biological function' includes those proteins with no recognized domains, as well as proteins with recognized, conserved domains of unknown function. The category 'transcriptional regulators and DNA binding proteins' includes recognized transcription factor families and chromatin binding proteins, that may or may not be involved in regulation.

The sets of genes described above were analyzed for their putative functions, as listed at The Arabidopsis Information Resource , using gene ontology searches  and published literature. Divisions into broad functional classes are shown in Figure 4. The proportions of the different categories vary little between the putative expression groups. The most prominent categories are proteins with unknown function and proteins involved in metabolism. There are also many putative transcription factors and DNA binding proteins (Table 2), which are good candidates for regulators of ovule development. The proportion of transcription factors is approximately 20%, which is higher than estimates for the proportion of transcription factors found in the genome (6–7%) [84-86].

**Table 2:** Identified transcriptional regulators and DNA-binding proteins.

A						
Clust	Gene	Gene Symbol	Description	WT E vs ANT E	WT E vs INO E	WT F vs INO F
(2)	At5g57390	AIL5	AP2/EREBP, ANT-like (organ size control, inflorescence)	-2.17	-1.33	-1.36
(3)	At1g79700	---	AP2/EREBP, AP2-like	-2.38	-1.37	-2.29
(5)	At2g46530	ARF11	auxin-responsive factor	-2.18	-1.19	1.05
(10)	At2g33860	ETT	auxin-responsive factor (flower development)	-2.37	1.03	1.07
(4)	At1g26680	---	B3 REM family	-2.86	-1.15	-1.10
(10)	At5g18090	---	B3 REM family	-2.06	-1.11	1.16
(7)	At3g46770	---	B3 REM family	-1.54	-1.26	-1.44
(11)	At3g61970	NGA2	B3 NGATHA family (lateral organ development, gynoecium)	-2.24	-1.04	-1.42
(7)	At2g20180	PIL5	bHLH family (light responsive GA synthesis repressor)	-3.13	-1.42	-2.00
(3)	At4g37610	BT5	BTB/POZ and TAZ zinc finger	-4.91	-1.37	-2.40
(0)	At3g48360	BT2	BTB/POZ and TAZ zinc finger (telomerase activation)	-4.78	-1.26	-2.59
(8)	At1g68640	PAN	bZIP family (floral organ nmber)	-2.16	-1.17	1.31
(2)	At3g55560	AGF2	DNA-binding At-hook family	-2.69	-1.16	-1.54
(10)	At4g24150	ATGRF8	growth-regulating factor family	-2.70	-1.10	-1.13
(9)	At1g76110	---	HMG1/2, ARID/BRIGHT DNA-binding domain	-3.01	-1.20	-1.14
(4)	At1g04880	---	HMG1/2, ARID/BRIGHT DNA-binding domain	-2.35	1.07	1.06
(7)	At4g36740	ATHB40	homeobox-leucine zipper Class I family	-3.80	-1.89	-4.73
(11)	At1g75430	---	homeodomain protein	-2.02	1.03	1.04
(10)	At5g41410	BEL1	homeodomain protein (ovule development)	-2.66	-1.12	-1.11
(7)	At5g17300	---	myb family	-2.01	-1.21	-1.91
(3)	At4g37260	MYB73	myb R2R3 family	-1.86	-1.03	-1.58
(0)	At5g51910	---	TCP family	-1.52	-1.16	-1.42
(11)	At2g01500	PFS2	WUS type homeobox (ovule development)	-2.17	-1.50	-1.59
(8)	At1g69180	CRC	YABBY family (abaxial cell development)	-2.19	1.02	-1.18
(7)	At2g36320	---	zinc finger (AN1-like) family	-1.51	-1.06	-1.37
(7)	At5g57660	---	zinc finger (B-box type) family	-1.76	-1.13	-1.66
(3)	At2g25900	---	zinc finger (CCCH-type) family	-2.23	-1.07	-1.79
(7)	At5g61120	---	zinc finger (PHD type) family	-2.02	-1.23	-1.18

B						

Early	At5g61590	---	AP2/EREBP, ERF subfamily B-3	-1.85	-1.63	-2.48
Full	At2g18050	HIS1-3	histone H1-3 (drought stress inducible)	-1.89	-1.34	-4.50
Full	At2g18550	ATHB21	homeobox-leucine zipper Class I	-2.13	-1.41	-3.66
Full	At5g03790	ATHB51/LMI1	homeobox-leucine zipper Class I (LFY target, meristem identity)	1.58	-2.23	-6.65
Early	At3g54340	AP3	MADS-box protein (floral development)	-1.82	-2.38	-6.38
Full	At5g01840	AtOFP2	ovate family, interacts with BLH4 (transcriptional repressor)	-1.14	-1.13	-2.34
Early	At2g40750	WRKY54	WRKY family transcription factor (defense response)	-1.59	-2.10	-1.58
Early	At5g06070	RBE	zinc finger (SUP-like C2H2 type) family	-2.30	-2.01	-1.98

C						

	At5g18000	REM18	B3 family, reproductive meristem (regulated by STK/SHP1/2)	-3.37	-1.51	-1.44
	At5g42630	ATS/KAN4	GARP family transcription factor (integument development)	-7.13	-2.00	-1.31
	At3g56400	WRKY70	WRKY transcription factor (plant senescence, defense)	-2.66	-1.74	-2.05
	At1g23420	INO	YABBY transcription factor (integument development)	-10.33	-4.04	-9.14
	At1g68190	---	zinc finger (B-box type) transcription factor	-2.28	-1.67	-2.03
	At3g23130	SUP	zinc finger (C2H2 type) (floral development)	-2.03	-1.40	-1.55

Transcription regulators and DNA binding proteins predicted to be expressed in the inner integument, ovule primordia and medial regions (A), the outer integument (B) and both integuments (C). The listed genes show strong evidence of expression in the integuments (2-fold decreased in one mutant or significantly decreased in both mutants). Fold changes between pair-wise comparisons are given (negative values indicates a lower value in the mutant). SOM cluster assignment or evidence stemming from the EARLY or FULL arrays are noted.

Within the set of transcriptional regulators, several families are represented, including different types of Zn finger (9), B3 (5), homeodomain leucine zipper (3), myb (2), bZIP (1), HMG/ARID (2), MADS (1), bHLH (1), YABBY (2), homeodomain (3), ANT-like (2), WRKY (2), ARF (2), ERF (1), TCP (1), and GARP/KANADI (1) proteins [84]. These encompass both characterized and uncharacterized proteins, and make good candidates for ovule development regulators. Groups of gene family members form good targets for analysis, as these genes, if they act redundantly in ovule development, would not be found through mutant screens.

Of the five identified B3 domain family proteins, four are part of the reproductive meristem (REM) family [87]. Two of the *REM *genes are very similar to each other (63% amino acid identity) and occur close to each other on Chromosome 5: At5g18000 (*REM18*) [88] and At5g18090. *REM18 *is regulated by the ovule identity complex formed by STK, SHP1/2 and SEP [76,88,89].

Most of the seventeen class I homeodomain leucine zipper proteins (HD-ZIP I) are uncharacterized. All 3 members of the δ subclass, *ATHB40 *(At4g36740), *ATHB21 *(At2g18850) and *ATHB53 *(At5g66700), show decreased expression in the mutants. This group of genes is expressed in inflorescences, and is induced by ABA and NaCl treatment in seedlings and ovules [90,91]. The related subclass ε contains two proteins *ATHB51 *(At5g03790) and *ATHB22 *(At2g36610). *ATHB51*/*LATE MERISTEM IDENTITY 1 *(*LMI1*), which was identified as a putative outer integument gene, is activated by LFY in meristems and regulates *CAULIFLOWER *expression and leaf/bract formation [92-94]. *ATHB22 *shows no evidence of expression in this experiment, but the MPSS database [24] shows low expression in inflorescences that drops to near zero in *agamous *inflorescences, implying carpel or stamen expression. No ovule mutant phenotypes were observed from putative insertional knockouts of *ATHB51/LMI1 *or *ATHB40 *(data not shown), and mutant combinations might be required to expose a role in ovule development, possibly as developmental regulators or environmental response factors.

Proteins with TAZ zinc fingers and BTB/POZ protein binding domains were shown to bind calmodulin and the BET class of chromatin binding and modification proteins that contain a bromodomain [95-98]. Two of these genes are predicted to be expressed in regions affected in the *ant *mutant and show almost identical expression profiles, being greater than 4-fold decreased in *ant *and 2.5-fold decreased in *ino*. BTB AND TAZ DOMAIN PROTEIN 2 (BT2) (At3g48360) induces telomerase activity in response to auxin [99], while BT5 (At4g37610) has no described function. Another pair of related genes, the HMG ARID transcription factors At1g76110 and At1g04880, are putative chromatin binding proteins [84] and putative inner integument or primordia genes. The specific functions of these genes are not known, and they represent interesting candidate genes for ovule development.

Several genes that encode proteins involved in protein modification and proteolysis were identified in this analysis. These include proteins involved in ubiquitin-mediated proteolysis, as well as RING proteins, protein kinases and proteases. A group of enzymes involved in trehalose metabolism, (4 of 11 trehalose-6-P synthase genes and a trehalase) were also identified. Trehalose synthesis has been shown to affect trichome morphology and plant architecture in Arabidopsis through regulation of cell shape [100], and could be acting similarly in the gynoecium.

### Validation of expression profiles and integument group predictions

The effectiveness of the array methodology and experimental design was evaluated using three approaches, quantitative RT-PCR (qRT-PCR), *in situ *expression analysis of select genes, and detection of known, expected genes within the integument groups as detailed above.

**Figure 5:**
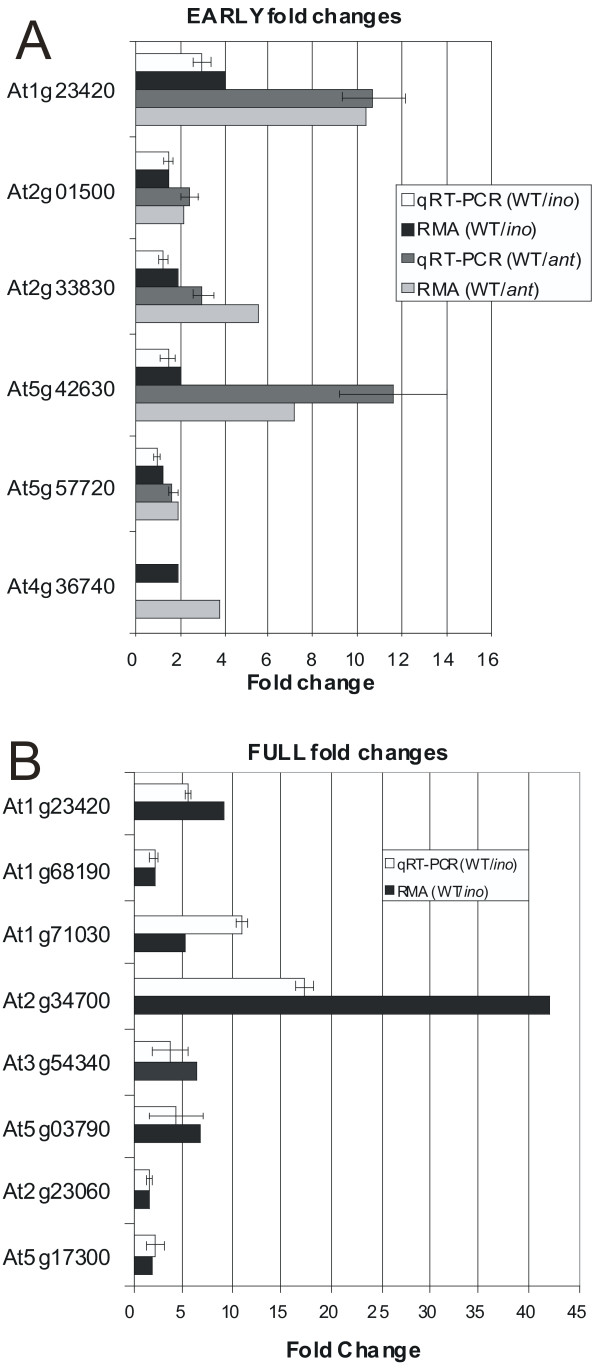
**Comparison of values obtained for differential expression using qRT-PCR and microarrays**. Relative expression levels obtained through qRT-PCR were compared with microarray expression levels (RMA derived) for selected genes. Error bars for qRT-PCR values are the standard deviations (n ≥ 3). (A) Comparisons between WT E and *ant *E, and between WT E and *ino *E. For the gene At4g36740 no amplification product was obtained from the mutants, indicating that mRNA for this gene was below the limit of detection using qRT-PCR. (B) Differential expression between WT F and *ino *F.

qRT-PCR was used to obtain an independent assessment of a sample of the microarray results [101], to test for spurious results due to cross hybridization, alternative splicing, or technical problems leading to inaccurate measurement of expression. Twelve genes that represented a range of fold changes and absolute expression levels were tested. The relative expression levels determined by qRT-PCR are compared with fold changes from the arrays in Figure 5, and for all the genes tested the direction of the fold change was confirmed, although there was variability in the magnitude of fold changes. When fold changes were small (< 2) the microarray and qRT-PCR results were more similar than when fold changes were larger. The rankings of genes by fold change did not vary widely between the two methods, so that relative differences in expression levels were also confirmed by the qRT-PCR method.

**Figure 6:**
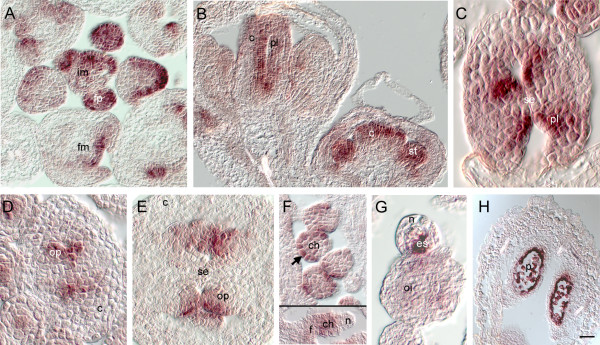
***In situ *hybridization pattern of At3g55560 in inflorescences**. (A – F): anti-sense probe; (G, H): sense probe. (A) Transcripts of At3g55560 were detected in the outer cell layers of floral primordia and floral organ primordia, and were maintained in the medial region of the elongating carpel (B). (C, D) Expression specific to the placenta was observed prior to fusion of the septum. (E) Emerging ovule primordia showed specific expression, that was maintained most strongly in the outer cell layers of the chalaza as the ovules developed (F). Expression was maintained at low levels in the distal funiculus and chalaza as the integuments initiated. (G) The sense probe detected RNA in the megaspore mother cell and developing embryo sac, as well as in the tapetum and pollen at maturity (H), but not in the structures that hybridized to the anti-sense probe. Bar = 20 μm in A, B, and F (bottom); 5 μm in C, and G; 10 μm in D, E, and F (top); 40 μm in H. c, carpel; ch, chalaza; es, embryo sac; f, funiculus; fm, floral meristem; fp, flower primordium; im, inflorescence meristem; n, nucellus; oi, outer integument; op, ovule primordium; p, pollen; pl, placenta; se, septum; st, stamen.

Analysis of mRNA expression patterns with *in situ *hybridization tests the predictive value of the expression profiling groups and provides important information for understanding gene function. *In situ *hybridizations were performed for At3g55560 (*AT-HOOK PROTEIN OF GA FEEDBACK 2*, *AGF2*), that encodes an At-hook DNA binding protein [102]. This gene showed a 2.7 fold decrease in *ant *relative to wild type, and a slight decrease (1.5 fold) in *ino *in the FULL arrays, and was in cluster 2, predicting expression in primordia, medial regions or inner integument with later embryo sac or outer integument expression. In confirmation of this prediction, early expression was seen in the placenta and ovule primordia, as well as the inflorescence meristem and flower primordia, and in the outer integument and distal funiculus of the ovule later. Serial sections indicated that expression was highest in the anlagen and primordia in the outermost 2 to 3 cell layers of flower primordia (Figure 6A). Expression was observed in the floral organ primordia, and persisted in growing carpels, stamens and petals (Figure 6B). The petal expression was highest in the edges of the petals, and expression in the anthers was highest in the center of each locule, prior to pollen formation. After microsporogenesis, expression in the tapetum and pollen decreased and was undetectable at maturity (not shown). In carpels, expression was limited very early to the parietal placental regions, before fusion of the septum (Figure 6C). Expression remained high in the ovule primordia as they formed as protrusions from the placenta (Figure 6D, E), and localized to the distal funiculus and outer integument after integument initiation (Figure 6F). By maturity, expression could not be detected in any part of the ovule (data not shown). A sense probe made from the same construct showed a distinct pattern confined to sporogenous cells, with a high level of expression seen in the tapetum and pollen and in the developing embryo sac (Figure 6G). In addition, MPSS signatures exist in this genomic region for this strand which have a different distribution pattern from the signatures for the coding strand [24].

**Figure 7:**
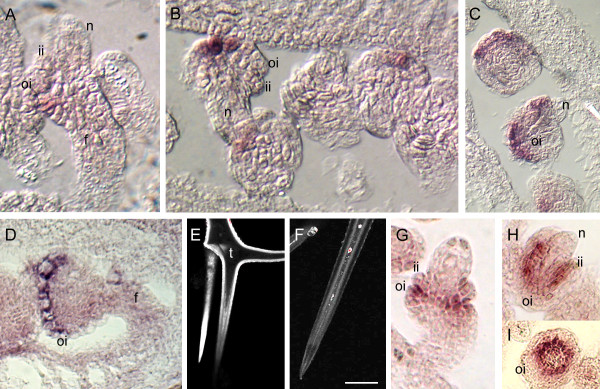
***In situ *hybridization pattern of At2g34700 and *ATS *in ovules and sub-cellular localization of At2g34700-GFP protein fusion**. (A-D): At2g34700 antisense probe; (E, F): confocal fluorescence micrographs of trichomes; (G – I): *ATS *antisense probe. Expression of At2g34700 was seen in the basal outer integument at emergence, (A) and was often in only one to two cells in the outer cell layer of the outer integument (B). As the integument grew, expression expanded both basally and apically but was not present in all cells of the outer layer at the same time (C). Close to anthesis, expression was strong and uniform in the outer integument (D). Arabidopsis trichomes stably transformed with a construct constitutively expressing a C terminal GFP fusion protein (P-UBQ10: At2g34700-GFP) showed fluorescence predominantly in the cell wall, indicating secretion of the fusion protein (E). Untransformed wild type trichomes showed low autofluoresence in the cell, mostly at the cell wall-plasma membrane junction (F). Excitation was increased several fold relative to (E) in order to observe the dimmer fluorescence. As the two integuments emerged, *ATS *expression was seen in the outer (abaxial) layer of the inner integument, and inner (adaxial) layer of the outer integument (G), and became confined to the inner integument as it extended along the nucellus (H). This expression formed a ring corresponding to inner integument encircling the nucellus (I). Scale bar = 30 μm in A, B, D, G, and H; 45 μm in C and I; 25 μm in B and E; 30 μm in E; and 25 μm in F. f, funiculus; n, nucellus; ii, inner integument; oi, outer integument; t, trichome.

*In situ *hybridization was also performed for At5g42630 that had shown a 7-fold decrease in *ant *and a 3.5-fold decrease in *ino *relative to wildtype. These array results, that predicted expression in both integuments, were used in combination with a separate map-based cloning effort (that had narrowed the search to fourteen candidates) to identify this gene as *ABERRANT TESTA SHAPE *(*ATS*) [103]. Full characterization of *ATS *is published elsewhere [104]. The *ats *mutant is affected in both integuments, and *in situ *hybridization showed initial expression in both integuments that subsequently resolves to expression in the inner integument, confirming the prediction from the array results (Figure 7G – 7I) [104].

The gene with highest fold change observed between wild type and *ino*, At2g34700, was categorized as an outer integument gene, (Figure 3C). *In situ *hybridization confirmed this result, showing that this gene is expressed solely in the outer integument in inflorescences, and likely in the outermost layer of the outer integument (Figure 7A – 7D). MPSS data for this gene indicates that this protein is limited to expression in inflorescences and therefore is likely to be an outer integument-specific gene. The protein product of At2g34700 is similar to the pollen Ole e 1 allergen from olive [105] and is a member of an uncharacterized group of plant-specific proteins that are likely secreted and may act as extensins. Expression was first visible in the basal/proximal region of the outer integument (Figure 7A) and initially localized to only one or two cells (Figure 7B). Expression spread to most cells of the outer cell layer as the outer integument grew (Figure 7C) and close to maturity, expression was retained at high levels in the outer cell layer (Figure 7D).

As the At2g34700 protein is predicted to be secreted, (TargetP 1.1) [106], subcellular localization was investigated using a C-terminal fusion of the GFP coding sequence to a cDNA encoding the At2g34700 protein, under control of the CaMV 35S promoter [107]. GFP was observed most strongly in trichomes and guard cells of transformants and in these cells the fluorescence was localized to the cell wall (Figure 7E). Two Ds transposon insertion lines [108] were obtained and lines containing homozygous insertions were identified using PCR. Neither line showed any differences in phenotype from wild type when examined with SEM (data not shown). There are two similar genes (63% amino acid similarity) in the Arabidopsis genome that could be redundant with At2g34700, alternatively, loss of function of this gene may not produce noticeable effects under normal conditions.

## Discussion

In this study, the spatial and temporal expression of genes in Arabidopsis integuments was analyzed by comparing the gene expression profiles of ovule morphogenesis mutants. The grouping of genes into broad domains of expression had predictive power, as shown by the correct assignment of genes with know expression patterns and the results of *in situ *hybridizations performed on candidate genes. At least thirty uncharacterized genes encoding proteins with likely regulatory function were identified in this study as having preferential expression in integuments. Thus, the use of mutants that lack specific structures to identify gene expressed in those structures was successful in providing new candidates that are likely to be playing roles in integument growth and development.

The candidate genes were selected on the basis of a combination of robust statistical tests and biological information. Approximately 4000 differentially regulated genes were initially identified by pair-wise statistical tests, for which the FDR rate was kept at 1%, which implied that approximately 40 genes were incorrectly identified as significant. Subsequently, the sets of genes were subjected to biological tests, to ensure that their expression levels were logical, given the nature of the mutants: genes had to show a decrease in expression in the mutants, and, where necessary, in both mutants. Next, the genes were analyzed for shared patterns of expression and sorted into groups using known expression patterns as profile indicators. On the basis of these indicators and to bring the number of genes to a manageable level, the groups were further subjected to a filter whereby genes were only retained if they were greater than 2-fold changed or had significant q-values in two pair-wise comparisons, resulting in a set of 207 genes. The use of a fold-change cutoff was justified by the observation that more specific expression patterns had higher fold changes in this experimental context, helping to differentiate between broadly expressed genes and those with ovule-specific expression. In addition, genes with higher fold changes were more likely to be selected by different statistical tests, giving more confidence to their selection.

There was a small set of twenty-five genes (including six putative transcription factors) that were predicted to be expressed predominantly in both integuments, including the gene *ATS/KAN4*, now known to be expressed in both integuments [104]. Approximately ten-fold that number were predicted to be predominantly expressed in the outer integument (including at least ten putative transcription factors), and 50 of these genes were decreased by more than two fold in *ino*. Known outer integument genes were identified, such as *AP3*, and an unknown gene was shown to be expressed in the outer integument using *in situ *hybridization (At2g34700). Some genes, such as *RBE *and At4g12960, have described expression patterns that differ from the predicted expression from the array, with expression in both integuments instead of just one or the reverse. Taken together, these results show that the array analysis was successful at predicting overall expression in the integuments and for most genes can predict whether that expression is in the outer or both integuments. Several genes (132 with 2-fold decrease in *ant *or significant decrease in *ino *F) were identified as candidates for early inner integument expression. While the aim of this study was to uncover novel genes involved in integument development, the data have also shown a clear ability to identify those genes expressed in the placenta and ovule primordia, as shown by *in situ *hybridization of the gene *AGF2/*At3g55560. This is a useful result as there is much that remains mysterious about placenta formation and the initiation of ovule primordia. Clustering of the different expression profiles suggests pattern differences between integument and placental expression, but further expression characterization will be necessary to sort such genes from those expressed in the inner integument.

Separately pooling early and late stages decreased the complexity of the samples and allowed better resolution of expression differences of lower expressed genes and genes active early in ovule development that may be key regulators of developmental processes. In addition, gene expression changes due to the failure of the embryo sac to develop were reduced with the earlier samples, as there were fewer putative gametophyte genes in the EARLY samples than the FULL samples. Although fifty of the 207 selected genes have evidence of embryo sac expression from other array experiments, there is a significant probability that such genes are not only expressed in the gametophyte, as genes such as *PFS2, RBE*, At2g34700 and At4g12960 have been shown to have specific integument expression, despite their identification as putative embryo sac genes [30,73,78]. This work has also confirmed that the sensitivity of the arrays was sufficient to detect changes in integument expression even within the complex tissue of whole pistils. A similar ability to distinguish differentially expressed genes was observed in the comparison of whole siliques of wild type and heterozygous *medea *mutants that showed 50% embryo abortion [32], and by comparison of pistils with and without gametophytes [34]. This sensitivity, however, was challenged when genes were expressed at lower levels or in very few cells, as seen with the meiosis and gametophyte cell specific genes, which are very likely not expressed in the *ant *mutant and yet show no difference between the mutant and wild type. This was partly beneficial, as these genes would contaminate the desired set of integument genes. However, if some genes were expressed very transiently in the integuments or in only a few cells, these genes would be unlikely to be found. The use of a relatively high fold change cutoff would also reduce the probably of finding such genes. Fortunately, genes expressed in integument primordia may not be affected as the primordia comprise several cells due to the ring or half ring nature of the integuments. Many of the identified genes were not at putatively absent levels in the mutants implying expression in other regions of the pistil. This is important because there is evidence that regulators of integument development have other roles in the carpel, as is the case for the *SHP *genes and *ANT *itself. However, the ability to detect different levels of expression was negatively affected when the expression was widespread, meaning that genes with more specific expression in integuments or primordia were preferentially identified.

There was an overabundance of transcription regulator genes in the dataset (20%) compared with the genome sequence (6–7%) [84-86]. While there is some uncertainty in such analyses due to evolving notions of transcription regulation, transcription factors appear to be selectively identified in this analysis. One reason for this could be the nature of the comparison that was being made. Since a large part of the cell types making up the samples were in common between the genotypes, with only specific structures being absent, more ubiquitously expressed genes such as metabolic enzymes are less likely to have been identified. Rather, those genes that have more specific expression patterns were identified, and transcription factors are often among these types of genes. The forty-two putative transcription factors identified at high fold change in this experiment occur in several gene families. It was surprising that only one MADS domain protein was identified at greater than 2-fold changed, as these genes are significantly involved in reproductive development. For some specific genes, such as *SHP2*, this is may be because of their more general expression in the carpels.

Transcription profiles of gene family members can be compared to yield information on their possible redundant action, or to identify members that act alone in specific cell types. The four members of the HD-ZIP I family identified in this analysis display differences in expression profile, which will help to predict which genes to analyze in mutant combination. Similarly, *NGA2 *gene expression is decreased in *ant *by greater than 2-fold, which indicates that this family member is regulated differently from the remaining three *NGA *genes. *NGA *genes are thought to act redundantly in lateral organ growth [109], and *NGA2 *could be acting more specifically in the carpel medial regions or inner integument.

The confirmation of expression using *in situ *hybridization has provided useful information about two genes. The presence of the At2g34700 mRNA in the growing outer integument in a specific pattern, secretion of the At2g34700 protein into the cell wall, and the encoded extensin motifs suggest at least two possibilities for this gene. The protein may be acting in cell expansion or maturation, as the outermost cells of the outer integument are relatively large, and also undergo cell wall rearrangements after fertilization to accommodate secretion of mucilage [110]. Extensins are also implicated in defense responses, and many respond to wounding or other environmental signals. An accumulation of this protein in the cell wall could be part of the complex set of defenses that are put in place to protect the developing seed. As putative knockouts in this gene did not show any unusual ovule morphology, double or triple mutants with paralogs may be needed to determine function.

The expression of the gene At3g55560 (*AGF2*) in carpels was specific to the placental regions and early ovule primordia, while the MPSS and GeneAtlas (Genevestigator) [111] databases indicate that this gene is not limited to expression in the carpel, but is found in seedlings, leaves and roots, with strongest expression in callus. AGF2 contains an AT-hook motif thought to bind AT-rich regions of DNA [112], and that has been implicated in plants in binding to matrix attachment regions of chromosomes during mitosis [113,114] as well as binding to promoters as part of HMG transcription complexes [115]. AGF1 and AGF2 bind to the *GA3ox1 *promoter in vitro, and AGF1 has been shown to function in the GA-negative feedback regulation of that gene [102]. There is no additional evidence that AGF2 functions in GA signaling, but it is possible that this protein could be acting to control GA induced development in the placenta. One of three gibberellin receptors (GID1C) [116,117] is also identified as putatively expressed in medial regions or ovule primordia indicating a possible specific action of a set of giberellin regulators in ovule development.

Several auxin responsive genes and genes involved auxin transport and perception were among the set of genes that were considered decreased in the mutants. These were genes such as *PIN1*, which had previously been shown to be expressed in the ovule epidermis and integuments in a polarized manner [66]. Such expression is thought to result in the observed foci of auxin accumulation in growing regions such as the integument tips. It therefore is not surprising that three of the auxin receptor proteins (*TRANSPORT INHIBITOR RESPONSE 1 *(*TIR1*), *AUXIN SIGNALING F-BOX 1 *(*AFB1*) and (*AFB3*) [118-120]) are predicted to be expressed in regions affected by *ant*. A pertinent question is whether there are specific auxin response factors (*ARF*) [121] that act in ovule development. Only 2 *ARF*s were retained after applying the final fold change thresholds, including *ETT *and *AUXIN RESPONSE FACTOR 11 *(*ARF11*). *ETT *has a known role in the auxin-mediated growth and development of the gynoecium, but no specific role has been demonstrated for *ARF11 *[122,123]. ARF18, the most similar protein to ARF11 [123,124] (70% identity over most of the protein), shares a similar expression profile to the *ARF11 *gene but with lower fold changes that were significant but not sufficient to be included in the final set. This pair of genes could also be acting redundantly in ovules to mediate auxin responses and affect cell divisions and differentiation.

Our studies provide numerous candidate genes to serve as targets for further analysis for their specific expression patterns and function through reverse genetics. In addition, this dataset provides further utility as a resource for information on genes of interest identified through other means and also provides an as yet uncharacterized set of genes that were upregulated in the two mutants examined.

## Conclusion

This work identified a set of approximately two hundred candidate genes expressed in the integuments through comparison of wild type to mutant ovules. The genes are predicted to have expression in the outer integument, both integuments or the inner integument and ovule primordia, and these predictions were confirmed by the presence of known genes in these groups, and through in situ hybridizations. Different analysis methods were compared, and RMA was considered most effective at reducing variance for low expressing genes (such as transcription factors). Genes identified with the limma modified t-test differed by up to 50% from those identified by the dchip fold change test, but the limma test was more effective at identifying known genes that differ between genotypes and thus this test was used for the analysis. The results showed that it was possible to use a mutant, *ant*, with broad effects on plant phenotype to identify genes expressed specifically in ovules, when coupled with predictions from known gene expression patterns, or in combination with a more specific mutant, *ino*. Groups of genes known to act in plant growth regulator pathways (such as auxin and giberellin) were identified, confirming the importance of such pathways in organ patterning and growth and providing an indication of which family members are acting specifically in ovules. The studies yielded an over-abundance of transcriptional regulators in the identified genes, which form a set of candidate genes for evaluation with reverse genetics.

## Methods

### Plant material growth conditions

Wild type plants were Landsberg *erecta *(Ler) ecotype, and the *ant-4 *and *ino-1 *mutants were in the Ler background. Plants were grown in 24 hour light at 19°C in growth chambers, using a 1:1 mixture of Premier Pro-Mix 'BX' potting soil (Premier Horticulture, Oceanside, CA) and vermiculite. Once germinated, plants were fertilized using a complete nutrient solution once per week [125]. All plants used in the array analysis were grown in a single growth chamber and flats of pots were rotated three times per week to different shelves and orientation to help ensure even growth conditions. Pots containing a particular genotype were placed randomly in flats. For each genotype and stage, pistils from more than 20 plants were collected over several days, during the same time period each day (10 AM – 1 PM), and pooled. Pistils were collected into tubes on dry ice and stored at -80°C.

### RNA extraction and array hybridization

Total RNA was extracted using the Qiagen RNeasy Plant kit (Qiagen, Valencia, CA). aRNA synthesis was performed using the Ambion MessageAmp kit (Ambion, Austin, TX) with biotin-11-CTP (Perkin Elmer, Boston, MA) and biotin-16-UTP (Roche, Indianapolis, IN). aRNA was fragmented following Affymetrix protocol and total RNA, aRNA and fragmented aRNA were checked for fragmentation and purity by gel electrophoresis and absorbance. Samples were hybridized to Affymetrix ATH 1 Genome Arrays (Cat # 900385; Affymetrix, Santa Clara, CA). All arrays were processed by the Core Facility in the UC Davis Department of Medical Microbiology & Immunology using an Affymetrix Hybridization Oven 640, the Affymetrix 450 Fluidics Station, and an Affymetrix GeneChip 3000 Scanner. The array data sets were named for the genotype (WT, *ino *or *ant*), replicate number and ovule stage pool (E, F, L). All array hybridization data were deposited in the ArrayExpress database with accession number E-MEXP-1920.

### Data Analysis

Raw CEL data was generated using MAS 5.0 (Affymetrix). An RMA measure of gene expression was calculated using the *affylmGUI *(*affy *and *limma*) package implemented for the Bioconductor project running in the R environment [50,51,126]. Perfect match values only were used with quantile normalization [127]. Raw CEL files were also loaded into dchip (v1.3 release date: 07/20/2005) and were normalized and modeled using the PM-MM and PM-only algorithms. Scatter plots of the log_2 _expression measures between a set of replicates processed with different methods and Pearson correlation coefficients were prepared using Excel 2003 (Microsoft, Redmond, WA).

For the statistical tests, *affylmGUI *was used to compute the moderated t-statistic [49,52], and the log fold changes. P-values were adjusted for multiple testing using the Storey q-value method [56]. For dchip, the PM-only expression values of sets of replicates were used to compare with other genotypes using the 'compare samples' function, with the following criteria: means separated by at least 20 and a fold change of 1.2 (using the lower bound of 90% confidence interval). SOM cluster analysis was carried out in GeneCluster 2.1.7 (Broad Institute), chosen as the number of likely patterns was low and it was not important to identify sub-clusters. The 12 clusters that result from SOM clustering of 800 inner integument genes are shown in additional file 5.

Genes were considered putatively absent in a sample if the average value was below 12, which was chosen by assessing the values of a set of putatively root specific genes [128] in the pistil samples (additional file 10).

### qRT-PCR

The RNA samples for RT-PCR were subjected to DNase treatment (Promega, Madison, WI) and digestion by two four-base cutter restriction enzymes to ensure complete digestion of any contaminating DNA. Two of the three biological replicates used for the microarrays were used in the reverse transcription and quantitative PCR. 1 μg of each RNA was used in reverse transcription reactions with either 3.75 units of Thermoscript (Invitrogen, Carlsbad, CA) or no reverse transcriptase as a -RT control, which was tested for contaminating genomic DNA in PCR reactions. 2 μl of a 1:40 dilution of the RT reactions were used in each quantitative PCR (qPCR) reaction. Primers were designed using the SYBR Green option of the Beacon 2.0 primer design software (Premier Biosoft, Palo Alto, CA) (Additional file 11). qPCR reactions were carried out using an iCycler (Biorad, Hercules, CA) and the following PCR reaction mix: 20 mM Tris pH 8.4, 50 mM KCL, 3 mM MgCl_2_, 4% glycerol, 20 nM fluorescein diacetate, 0.5× BSA (New England Biolabs, Beverly, MA), 1:50 000 diluted SYBR GREEN I (Cambrex Bio Science, Rockland, ME), 0.2 mM each dNTP, 0.24 μM each primer and 0.6 U iTaq (Biorad, Hercules, CA).

The fluorescence threshold at which the cycle number (C_t_) was calculated was set at 25 CF RFU (curve-fit relative fluorescence units) for all experiments, close to the automatically determined threshold for each plate. The 60S ribosomal protein RPL14B gene (At4g27090) was used as a reference and showed very similar C_t _values (range: 19.17 – 19.70) in all sample types tested. The relative starting quantity of cDNA for a particular gene was determined in GENEX (Biorad) using the following equation: relative quantity = efficiency ^(control Ct-experimental Ct) ^based on Livak [129] and Vandesompele [130]. The mean PCR efficiencies of the primer sets were determined using LinRegPCR [131], using a linear regression model.

### Plasmids

Plasmids for production of probes for *in situ *hybridization were constructed as follows. The 1 kb At3g55560 coding region was amplified from Columbia genomic DNA using c55560F1: AGAATGGCGAATCCTTGG and c55560R1: CTAATCAATACGAAGGAGG and cloned into the pCR4-TOPO vector (Invitrogen, Carlsbad, CA) to form pDS148. The At2g34700 cDNA was amplified from the cDNA clone U20928 [132] using the primers c34700F3: ATACTAGTAATGGGTCTGGTAACAAAAGCTC adding the restriction site *Spe*1 and c34700R4ns: ATAGGATCCGTCTTCCAAGAGCACAGGCAGGCTC which removes the STOP codon, and adds the restriction site *Bam*H1. This fragment cut with *Bam*H1 and *Spe*1 was subcloned into pLitmus28 (New England Biolabs, Beverly, MA) cut with the same enzymes to form pDS137. This fragment was also cut and used in a three-way ligation with pLitmus28 cut with *Spe*1 and *Sac*1, and the GFP sequence subcloned from the pGFP1.1.5 plasmid [133] using *Bam*H1 and *Sac*1. The resulting plasmid, pDS138.3, was digested with *Spe*1 and *Sac*1 and the GFP-fusion fragment inserted into pMON999 [79] cut with *Xba*1 and *Sac*1 to give pDS142. This formed a sequence verified expression cassette using the 35S promoter driving expression of a chimeric gene that encodes a C-terminal fusion of the GFP protein to the At2g34700 protein. This plasmid was tested for transient expression by blasting into onion cells as described previously [134]. The resulting fusion protein formed aggregates of protein that were likely not localized correctly. Therefore, this plasmid was subcloned using *Not*1 sites into a transformation plasmid, pMLBART, forming pDS146. This clone was transferred using three-way mating into the Agrobacterium strain ASE and transformed into wild type Arabidopsis (Ler) plants.

### In situ hybridization

Wild type *Ler *inflorescences were fixed in FAA: formaldehyde (10%), ethanol (50%) and acetic acid (5%) overnight at 4°C and embedded in Paraplast Xtra (Electron Microscopy Sciences, Philadelphia, USA). Probe preparation and in situ hybridization were performed using a modification [135] of the protocol of Ferrándiz et al. [136]. Digoxigenin-labeled RNA probes were prepared from the clones above, linearized with appropriate enzymes and transcribed with T3 or T7 RNA polymerases. As a control for all *in situ *hybridization experiments, an antisense probe for the *INO *gene was used simultaneously. The *INO *hybridizations confirmed the expression pattern reported previously [35,79].

### Microscopy

Mutant and wildtype pistils were fixed for scanning electron microscopy (SEM) as described [137], using 5% glutaraldehyde with postfixation in 2% osmium tetroxide. Pistils were dissected following critical point drying to allow observation of ovules. SEM images were collected using a Hitachi S3500N microscope and processed using Photoshop v. 7.0.

For callose staining of embryo sacs, wild type and mutant pistils at anthesis were preprocessed by cutting the pistil just below the style, and at the base to allow entry of the stain, and immersed in 65°C 5 M NaOH for 5 minutes. Pistils were rinsed with water, stained with decolorized aniline blue for 2 hours and examined with fluorescence microscopy using a UV laser on a Zeiss (Oberkoche, Germany) Axioplan microscope and images were acquired with a MDS290 digital camera (Kodak, New Haven, CT) and edited in Photoshop v. 7.0 (Adobe, San Jose, CA).

Transgenic plants expressing the At2g34700-GFP fusion protein and wild type non-transgenic plants were examined on an Olympus (Orangeburg, NY) Confocal FV1000 microscope and digital images obtained with the integral Olympus camera and edited in Photoshop v. 7.0 (Adobe, San Jose, CA).

## Authors' contributions

The studies were conceived and planned by DJS and CSG. DJS performed the experimental studies and wrote the draft manuscript in consultation with CSG. The manuscript was edited and prepared for submission by DJS and CSG. Both authors approved the final manuscript.

## Supplementary Material

Additional file 1 **Scatterplots comparing methods of normalization and expression summarization**. A pair of hybridization replicates, *ant *1 E and *ant *2 E, were chosen to illustrate differences in data distribution after processing with different methods to produce a final expression measure for each gene. All values are log_2 _transformed and *ant *1E gene values are plotted on the y-axis against *ant *2 E on the x-axis. The red line indicates no difference in expression level between replicates, and the black lines indicate 2-fold changes between the replicates. Larger numbers of points far from the red diagonal indicate less correspondence between the replicates. Results were similar for a separate set of replicates (not shown). (A) RMA; (B) dchip perfect match (PM) only; (C) MAS 5.0; (D) dchip perfect match minus mismatch (PM-MM). Click here for file

Additional file 2 **Pearson correlation coefficients between replicates, comparing different processing methods**. Comparisons for all replicates between processing with RMA, dchip perfect match (PM) only, dchip perfect match minus mismatch (PM-MM), and MAS 5.0. Higher values indicated better correlation between replicates. Click here for file

Additional file 3 **Overlap between genes identified as significantly changed between mutant and wildtype using dchip and RMA-*limma***. (A) WT E vs *ant *E; (B) WT F vs *ino *F. Click here for file

Additional file 4 **Fold change values for genes known to be expressed in ovules as determined with two different analysis methods**. Fold changes are WT/mutant, with numbers > 1 indicating a decrease in the mutant. NI indicates those genes that were not selected by the test indicated. Click here for file

Additional file 5 **SOM clusters for 800 genes significantly decreased in *ant *E arrays compared with both WT E and *ino*E**. Clustering using SOM principles with an input of 12 clusters leads to the groups shown. The number of genes contained within each cluster is indicated in each box. The z-transformed (mean = 0; standard deviation = 1) gene expression values form the y-axis of each graph. Each black dot represents the mean expression level of all the genes in the cluster for an array. The order of the arrays is EARLY arrays first, with three WT followed by three *ino *and three *ant*. The FULL arrays are next (two WT followed by two *ino*) and the WT LATE arrays are listed last. The red lines show the upper and lower ranges of the expression values for the genes within the cluster. Click here for file

Additional file 6 **Selected genes with known carpel expression patterns identified by the arrays**. Genes are grouped by the similarity of their expression profiles in the arrays, and the published expression pattern in pistils is described. Click here for file

Additional file 7 **Genes predicted to be expressed in the inner integument, ovule primordia and/or medial regions**. Listed genes showed good evidence of expression in the indicated ovule regions on the basis of being either 2-fold decreased in the relevant mutant or showing a significant decrease in more than one mutant:wild type comparison. The fold changes between the pair-wise comparisons are given (natural scale) and a negative value indicates that the mutant value was less than the wild-type value. Genes are organized by broad functional categories, and for inner integument genes the cluster to which they were assigned is noted. For outer integument genes, the evidence of expression was from the EARLY or FULL arrays as noted. The * column shows whether a gene was putatively absent in any mutant and whether a gene was also identified in the analyses by Yu [33], Johnston [34] and Tung [31]. (**a**: mutant level less than 3.58 (log_2_); **es**: embryo sac; **tt**: transmitting tract; **s**: stigma). Click here for file

Additional file 8 **Genes predicted to be expressed in the outer integument**. As for additional file 7 Click here for file

Additional file 9 **Genes predicted to be expressed in both integuments**. As for additional file 7 Click here for file

Additional file 10 **Mean natural scale RMA values of putatively root specific genes in the 17 pistil arrays**. Table of genes used to estimate expression value for genes expected to be absent in the samples used. Click here for file

Additional file 11 **Genes tested with qRT-PCR and primers used**. Table of genes validated with qRT-PCR. Click here for file
