# Supplementary figures and images for: Expression-based discovery of candidate ovule development regulators through transcriptional profiling of ovule mutants
Source: BMC Plant Biol. 2009 Mar 16;9:29. doi: 10.1186/1471-2229-9-29 (PMC2664812; doi:10.1186/1471-2229-9-29)

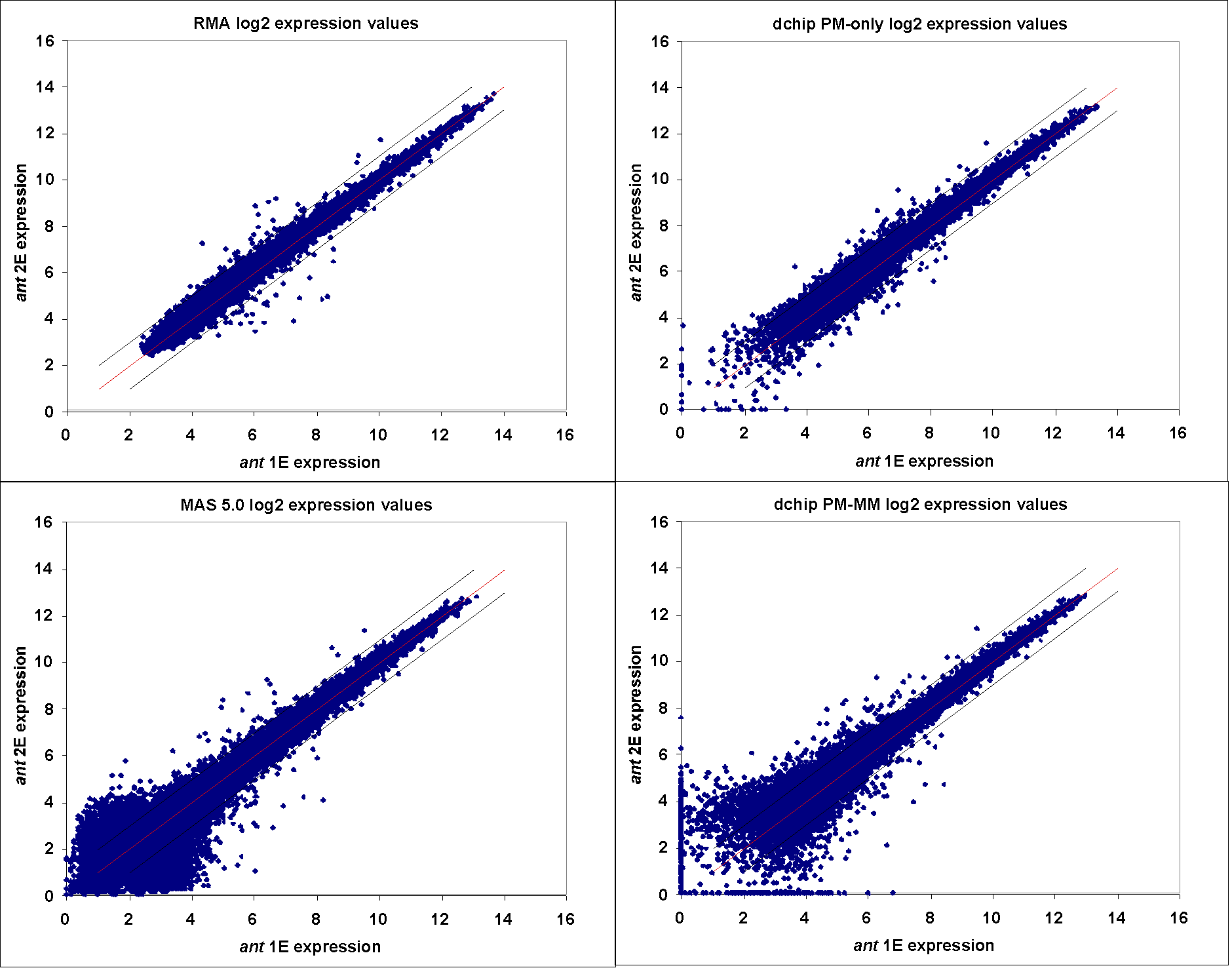

Supplement: Additional file 1 — Scatterplots comparing methods of normalization and expression summarization. A pair of hybridization replicates, ant 1 E and ant 2 E, were chosen to illustrate differences in data distribution after processing with different methods to produce a final expression measure for each gene. All values are log2 transformed and ant 1E gene values are plotted on the y-axis against ant 2 E on the x-axis. The red line indicates no difference in expression level between replicates, and the black lines indicate 2-fold changes between the replicates. Larger numbers of points far from the red diagonal indicate less correspondence between the replicates. Results were similar for a separate set of replicates (not shown). (A) RMA; (B) dchip perfect match (PM) only; (C) MAS 5.0; (D) dchip perfect match minus mismatch (PM-MM). [file 1471-2229-9-29-S1.tiff]

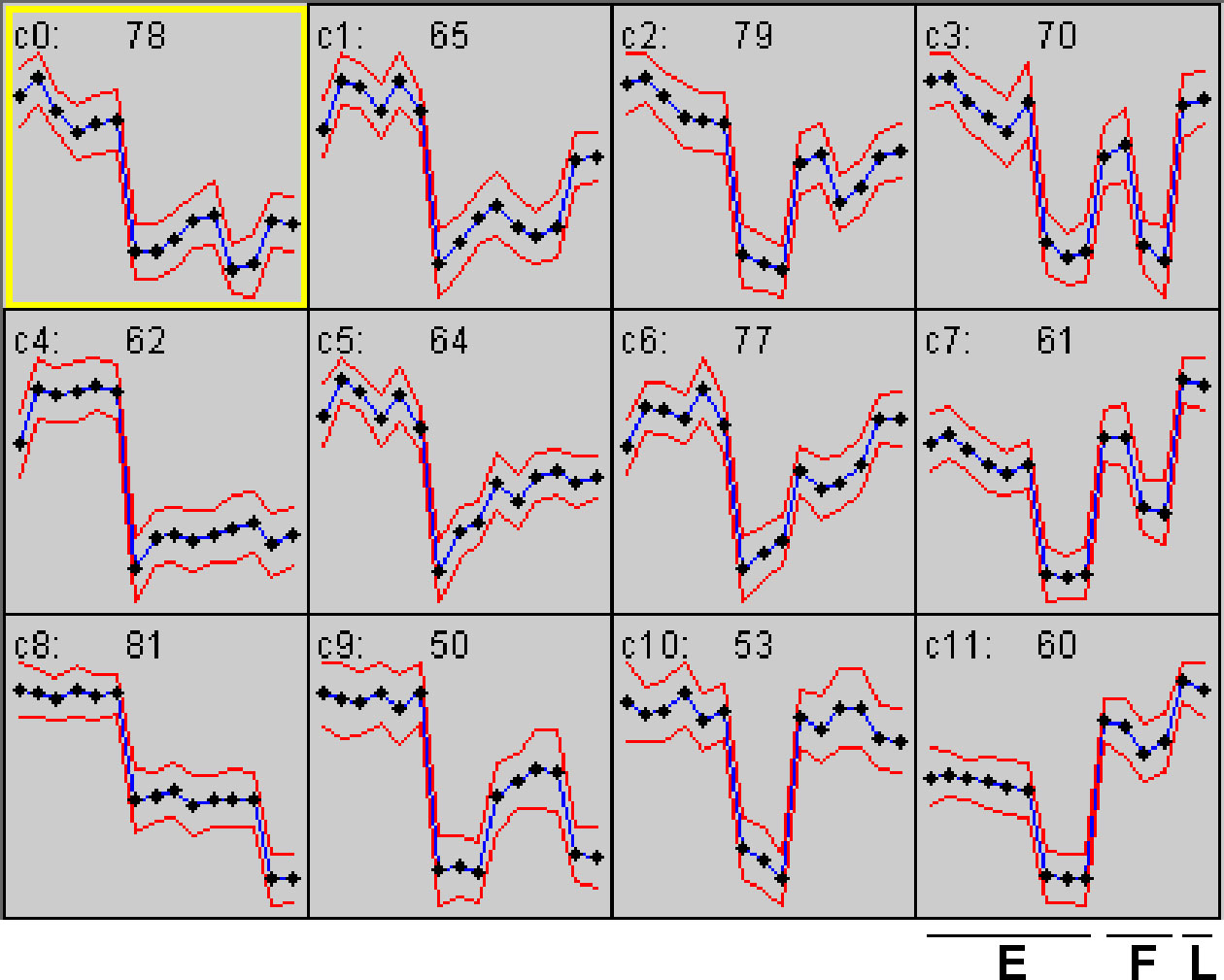

Supplement: Additional file 5 — SOM clusters for 800 genes significantly decreased in ant E arrays compared with both WT E and inoE. Clustering using SOM principles with an input of 12 clusters leads to the groups shown. The number of genes contained within each cluster is indicated in each box. The z-transformed (mean = 0; standard deviation = 1) gene expression values form the y-axis of each graph. Each black dot represents the mean expression level of all the genes in the cluster for an array. The order of the arrays is EARLY arrays first, with three WT followed by three ino and three ant. The FULL arrays are next (two WT followed by two ino) and the WT LATE arrays are listed last. The red lines show the upper and lower ranges of the expression values for the genes within the cluster. [file 1471-2229-9-29-S5.jpeg]
