# Supplementary material for: Expression-based discovery of candidate ovule development regulators through transcriptional profiling of ovule mutants
Source: BMC Plant Biol. 2009 Mar 16;9:29. doi: 10.1186/1471-2229-9-29 (PMC2664812; doi:10.1186/1471-2229-9-29)
Supplement: Additional file 2 — Pearson correlation coefficients between replicates, comparing different processing methods. Comparisons for all replicates between processing with RMA, dchip perfect match (PM) only, dchip perfect match minus mismatch (PM-MM), and MAS 5.0. Higher values indicated better correlation between replicates. [file 1471-2229-9-29-S2.pdf]

Additional file 2: Pearson correlation coefficients between replicates, comparing different processing methods.

Array Pair	RMA	dchip PM	dchip PM-MM	MAS5
ant: 1E vs 2E	0.9941	0.9848	0.9527	0.9879
ant: 2E vs 3E	0.9975	0.9953	0.9767	0.9940
ant: 1E vs 3E	0.9934	0.9827	0.9512	0.9865
ino: 1E vs 2E	0.9953	0.9922	0.9731	0.9912
ino: 2E vs 3E	0.9962	0.9950	0.9804	0.9924
ino: 1E vs 3E	0.9967	0.9934	0.9737	0.9932
WT: 1E vs 2E	0.9959	0.9913	0.9722	0.9907
WT: 2E vs 3E	0.9976	0.9954	0.9807	0.9956
WT: 1E vs 3E	0.9950	0.9873	0.9675	0.9897
ino: 1F vs 2F	0.9958	0.9932	0.9713	0.9942
ino: 2F vs 3F	0.9972	0.9937	0.9687	0.9949
ino: 1F vs 3F	0.9966	0.9938	0.9696	0.9950
WT: 1F vs 2F	0.9936	0.9888	0.9590	0.9921
WT: 2F vs 3F	0.9965	0.9930	0.9657	0.9947
WT: 1F vs 3F	0.9930	0.9878	0.9575	0.9876
**Average**	*0.9956*	*0.9912*	*0.9680*	*0.9920*
**Median**	*0.9959*	*0.9930*	*0.9696*	*0.9924*
