# Supplementary material for: Expression-based discovery of candidate ovule development regulators through transcriptional profiling of ovule mutants
Source: BMC Plant Biol. 2009 Mar 16;9:29. doi: 10.1186/1471-2229-9-29 (PMC2664812; doi:10.1186/1471-2229-9-29)

Additional file 3: Overlap between genes identified as significantly changed between mutant and wildtype using dchip and RMA-*limma.*
(A) WT E vs *ant* E; (B) WT F vs *ino* F

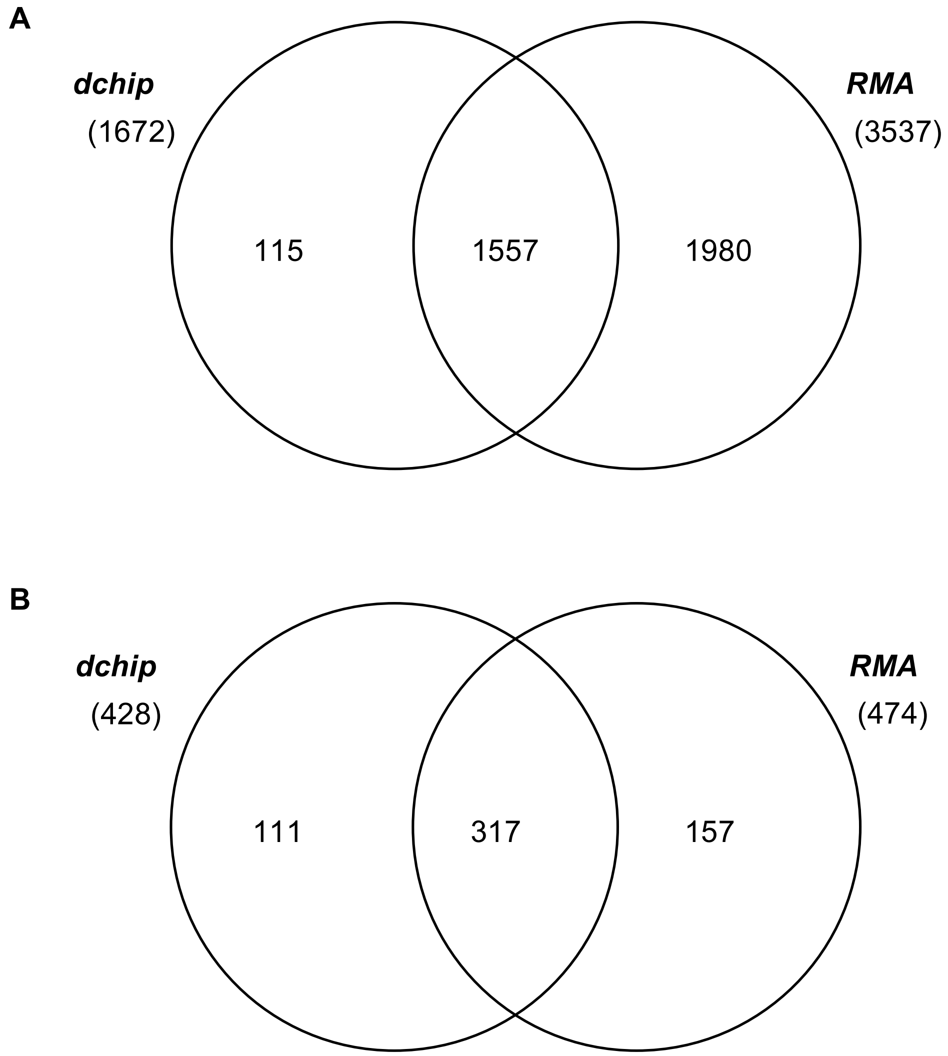

Supplement: Additional file 3 — Overlap between genes identified as significantly changed between mutant and wildtype using dchip and RMA-limma. (A) WT E vs ant E; (B) WT F vs ino F. [file 1471-2229-9-29-S3.pdf]
