# Supplementary material for: Expression-based discovery of candidate ovule development regulators through transcriptional profiling of ovule mutants
Source: BMC Plant Biol. 2009 Mar 16;9:29. doi: 10.1186/1471-2229-9-29 (PMC2664812; doi:10.1186/1471-2229-9-29)
Supplement: Additional file 4 — Fold change values for genes known to be expressed in ovules as determined with two different analysis methods. Fold changes are WT/mutant, with numbers > 1 indicating a decrease in the mutant. NI indicates those genes that were not selected by the test indicated. [file 1471-2229-9-29-S4.pdf]

Additional file 4: Fold change values for genes known to be expressed in ovules as determined with two different analysis methods.

			WT E/ant E		WT F/ino F		
**Name**	**Accession**	**Probe**	**dchip -LB**	**RMA-limma**	**dchip -LB**	**RMA-limma**	**Ref.**
ACR4	At3g59420	251521_at	NI	1.3			[1]
AIL5	At5g53790	247904_at	1.9	2.2			[2]
AP3	At3g54340	251898_at	NI	1.8	4	6.4	[3]
	At4g12960	254777_at	7.8	12	1.8	NI	
ATML1	At1g21750	254370_at	1.5	1.4			[4]
ATS	At5g42630	249206_at	3.7	7.1			[5]
BEL1	At1g75430	261111_at	NI	2			[6]
ERL1	At5g62230	247471_at	1.8	2			[7]
ERL2	At5g07180	250642_at	1.8	1.8			[7]
INO	At1g23420	262989_at	9.8	10.3	8.4	9.1	[8]
PDF2	At4g04890	255277_at	1.8	1.8			[9]
PFS2	At2g01500	266354_at	1.5	2.2	1.4	1.6	[10]
PHB	At2g34710	267316_at	1.6	1.6			[11]
PIN1	At1g73590	259845_at	1.6	1.6			[12]
RBE	At5g06070	250708_at	NI	2.3	NI	2	[13]
SHP2	At2g42830	263988_at	NI	1.3			[14]
SPT	At4g36930	246212_at	1.4	1.5			[15]

REFERENCES

1. Gifford ML, Dean S, Ingram GC: **The Arabidopsis *ACR4* gene plays a role in cell layer organisation during ovule integument and sepal margin development**. *Development* 2003, **130**:4249-4258.
2. Nole-Wilson S, Tranby TL, Krizek BA: ***AINTEGUMENTA-like* (*AIL*) genes are expressed in young tissues and may specify meristematic or division-competent states**. *Plant Mol Biol* 2005, **57**:613-628.
3. Hill TA, Day CD, Zondlo SC, Thackeray AG, Irish VF: **Discrete spatial and temporal cis-acting elements regulate transcription of the Arabidopsis floral homeotic gene *APETALA3***. *Development* 1998, **125**:1711-1721.
4. Lu P, Porat R, Nadeau JA, O'Neill SD: **Identification of a meristem L1 layer-specific gene in Arabidopsis that is expressed during embryonic pattern formation and defines a new class of homeobox genes**. *Plant Cell* 1996, **8**:2155-2168.
5. McAbee JM, Hill TA, Skinner DJ, Itzaki A, Hauser BA, Meister RJ, Reddy VG, Meyerowitz EM, Bowman JL, Gasser CS: ***ABERRANT TESTA SHAPE* encodes a KANADI family member, linking polarity determination to separation and growth of Arabidopsis ovule integuments**. *in preparation* 2005.
6. Reiser L, Modrusan Z, Margossian L, Samach A, Ohad N, Haughn GW, Fischer RL: **The *BELL1* gene encodes a homeodomain protein involved in pattern formation in the Arabidopsis ovule primordium**. *Cell* 1995, **83**:735-742.

7. Shpak ED, Berthiaume CT, Hill EJ, Torii KU: **Synergistic interaction of three ERECTA-family receptor-like kinases controls Arabidopsis organ growth and flower development by promoting cell proliferation**. *Development* 2004, **131**:1491-1501.
8. Villanueva JM, Broadhvest J, Hauser BA, Meister RJ, Schneitz K, Gasser CS: ***INNER NO OUTER*** **regulates abaxial-adaxial patterning in** ***Arabidopsis*** **ovules**. *Genes Dev* 1999, **13**:3160-3169.
9. Abe M, Katsumata H, Komeda Y, Takahashi T: **Regulation of shoot epidermal cell differentiation by a pair of homeodomain proteins in Arabidopsis**. *Development* 2003, **130**:635-643.
10. Park SO, Zheng Z, Oppenheimer DG, Hauser BA: **The** ***PRETTY FEW SEEDS2*** **gene encodes an Arabidopsis homeodomain protein that regulates ovule development**. *Development* 2005, **132**:841-849.
11. Sieber P, Gheyselinck J, Gross-Hardt R, Laux T, Grossniklaus U, Schneitz K: **Pattern formation during early ovule development in** ***Arabidopsis thaliana***. *Dev Biol* 2004, **273**:321-334.
12. Benkova E, Michniewicz M, Sauer M, Teichmann T, Seifertova D, Jurgens G, Friml J: **Local, efflux-dependent auxin gradients as a common module for plant organ formation**. *Cell* 2003, **115**:591-602.
13. Krizek BA, Lewis MW, Fletcher JC: **RABBIT EARS is a second-whorl repressor of AGAMOUS that maintains spatial boundaries in Arabidopsis flowers**. *Plant J* 2006, **45**:369-383.
14. Savidge B, Rounsley SD, Yanofsky MF: **Temporal relationship between the transcription of two Arabidopsis MADS box genes and the floral organ identity genes**. *Plant Cell* 1995, **7**:721-733.
15. Heisler MG, Atkinson A, Bylstra YH, Walsh R, Smyth DR: ***SPATULA*****, a gene that controls development of carpel margin tissues in Arabidopsis, encodes a bHLH protein**. *Development* 2001, **128**:1089-1098.
