# Supplementary material for: Expression-based discovery of candidate ovule development regulators through transcriptional profiling of ovule mutants
Source: BMC Plant Biol. 2009 Mar 16;9:29. doi: 10.1186/1471-2229-9-29 (PMC2664812; doi:10.1186/1471-2229-9-29)
Supplement: Additional file 6 — Selected genes with known carpel expression patterns identified by the arrays. Genes are grouped by the similarity of their expression profiles in the arrays, and the published expression pattern in pistils is described. [file 1471-2229-9-29-S6.pdf]

## Additional file 6: Selected genes with known carpel expression patterns identified by the arrays.

| Gene | Cluster | FC | Function | Expression Pattern | Ref. |
|---|---|---|---|---|---|
| **A: Expression early in ovule primordia, placenta and integuments** | | | | | |
| *NUB* | 4 | -1.6 | Lateral growth, adaxial carpel growth | Adaxial carpel, placenta, ovule primordia. | [1] |
| *CRC* | 8 | -2.2 | Carpel specification | Abaxial carpel, inner strand next to placenta. | [2] |
| *CUC2* | 8 | -1.7 | Organ separation | Placenta, between ovule primordia, septum, nucellus/chalaza boundary. | [3] |
| *ER* | 8 | -1.4 | Growth inhibitor signal | Early carpels, carpel walls, slightly in ovules | [4, 5] |
| *LFY* | 8 | -1.4 | Floral induction | In carpels? | [6] |
| *PAN* | 8 | -2.2 | Organ number | Early medial region, placenta, ovule primordia, slightly in later ovules | [7] |
| *SPT* | 8 | -1.5 | Growth of medial carpel regions | Medial region, ridge, septum, stigma, ovule primordia, integument tips, mmc | [8] |
| *PIN1* | 9 | -1.6 | Polar auxin transport | L1, carpel primordia, ovule primordia, integuments. | [9] |
| *ATML1* | 6 | -1.4 | L1 identity | L1 of meristems, placenta, ovule primordia, integument primordia. | [10] |
| *BEL1* | 10 | -2.7 | Chalazal specification | Chalaza and integuments. | [11] |
| *ATCEL2* | 10 | -1.7 | Cellulase, wall extension | Placenta, ovule primordia, septum | [12] |
| *ETT* | 10 | -2.4 | auxin response growth regulator | Abaxial carpel, replum | [13] |
| **B: Expression in carpel, ovule primordia, integuments or embryo sac** | | | | | |
| *MP* | 0 | -1.6 | TF mediating auxin signals | Center of ovule primordia, adaxial carpel wall, integuments not described. | [14] |
| *PHB* | 2 | -1.6 | Adaxial identity | Adaxial inner integument, carpel? | [15] |
| *ERL2* | 2 | -1.8 | Epidermal patterning | Early carpels, ovules, outer integument higher, style. | [5] |
| *PDF2* | 2 | -1.8 | L1 identity | L1 of carpel and ovule primordia, integuments not described | [16] |
| *AIL5* | 2 | -2.2 | ANT-like control of organ size | Medial ridge, placenta, ovule primordia, distal funiculus and outer integument. | [17] |
| **C: expression in carpel, ovule primordia or integuments late in development** | | | | | |
| *FDH* | 3 | -1.4 | Long chain lipids | Ovule primordia, integuments, later in chalazal pole. | [18] |
| *ERL1* | 11 | -2.0 | Epidermal patterning | Early carpel primordia, ovules. | [5] |
| *PFS2* | 11 | -2.2 | Repression of AG? | Placenta, ovule primordia, chalaza, integuments, nucellus. | [19] |

1. Dinneny JR, Weigel D, Yanofsky MF: **NUBBIN and JAGGED define stamen and carpel shape in Arabidopsis**. *Development* 2006, **133**:1645-1655.
2. Bowman JL, Smyth DR: ***CRABS CLAW*, a gene that regulates carpel and nectary development in Arabidopsis, encodes a novel protein with zinc finger and helix-loop-helix domains**. *Development* 1999, **126**:2387-2396.
3. Ishida T, Aida M, Takada S, Tasaka M: **Involvement of *CUP-SHAPED COTYLEDON* genes in gynoecium and ovule development in *Arabidopsis thaliana***. *Plant Cell Physiol* 2000, **41**:60-67.
4. Torii KU, Mitsukawa N, Oosumi T, Matsuura Y, Yokayama R, Whittier RF, Komeda Y: **The arabidopsis *ERECTA* gene encodes a putative receptor protein kinase with extracellular leucine-rich repeats**. *Plant Cell* 1996, **8**:735-746.
5. Shpak ED, Berthiaume CT, Hill EJ, Torii KU: **Synergistic interaction of three ERECTA-family receptor-like kinases controls Arabidopsis organ growth and flower development by promoting cell proliferation**. *Development* 2004, **131**:1491-1501.
6. Weigel D, Alvarez J, Smyth DR, Yanofsky MF, Meyerowitz EM: ***LEAFY* controls floral meristem identity in Arabidopsis**. *Cell* 1992, **69**:843-859.
7. Chuang CF, Running MP, Williams RW, Meyerowitz EM: **The *PERIANTHIA* gene encodes a bZIP protein involved in the determination of floral organ number in *Arabidopsis thaliana***. *Genes Dev* 1999, **13**:334-344.
8. Heisler MG, Atkinson A, Bylstra YH, Walsh R, Smyth DR: ***SPATULA*, a gene that controls development of carpel margin tissues in Arabidopsis, encodes a bHLH protein**. *Development* 2001, **128**:1089-1098.
9. Benkova E, Michniewicz M, Sauer M, Teichmann T, Seifertova D, Jurgens G, Friml J: **Local, efflux-dependent auxin gradients as a common module for plant organ formation**. *Cell* 2003, **115**:591-602.
10. Lu P, Porat R, Nadeau JA, O'Neill SD: **Identification of a meristem L1 layer-specific gene in Arabidopsis that is expressed during embryonic pattern formation and defines a new class of homeobox genes**. *Plant Cell* 1996, **8**:2155-2168.
11. Reiser L, Modrusan Z, Margossian L, Samach A, Ohad N, Haughn GW, Fischer RL: **The *BELL1* gene encodes a homeodomain protein involved in pattern formation in the Arabidopsis ovule primordium**. *Cell* 1995, **83**:735-742.
12. Yung MH, Schaffer R, Putterill J: **Identification of genes expressed during early Arabidopsis carpel development by mRNA differential display: characterisation of *ATCEL2*, a novel endo-1,4-beta-D-glucanase gene**. *Plant Journal* 1999, **17**:203-208.
13. Sessions A, Nemhauser JL, McColl A, Roe JL, Feldmann KA, Zambryski PC: **ETTIN patterns the *Arabidopsis* floral meristem and reproductive organs**. *Development* 1997, **124**:4481-4491.
14. Hardtke CS, Berleth T: **The Arabidopsis gene *MONOPTEROS* encodes a transcription factor mediating embryo axis formation and vascular development**. *EMBO J* 1998, **17**:1405-1411.
15. Sieber P, Gheyselinck J, Gross-Hardt R, Laux T, Grossniklaus U, Schneitz K: **Pattern formation during early ovule development in *Arabidopsis thaliana***. *Dev Biol* 2004, **273**:321-334.

16. Abe M, Katsumata H, Komeda Y, Takahashi T: **Regulation of shoot epidermal cell differentiation by a pair of homeodomain proteins in Arabidopsis**. *Development* 2003, **130**:635-643.
17. Nole-Wilson S, Tranby TL, Krizek BA: ***AINTEGUMENTA-like*** **(*AIL*) genes are expressed in young tissues and may specify meristematic or division-competent states**. *Plant Mol Biol* 2005, **57**:613-628.
18. Pruitt RE, Vielle-Calzada JP, Ploense SE, Grossniklaus U, Lolle SJ: ***FIDDLEHEAD*****, a gene required to suppress epidermal cell interactions in Arabidopsis, encodes a putative lipid biosynthetic enzyme**. *Proc Natl Acad Sci U S A* 2000, **97**:1311-1316.
19. Park SO, Zheng Z, Oppenheimer DG, Hauser BA: **The *PRETTY FEW SEEDS2* gene encodes an Arabidopsis homeodomain protein that regulates ovule development**. *Development* 2005, **132**:841-849.
