# Supplementary material for: Expression-based discovery of candidate ovule development regulators through transcriptional profiling of ovule mutants
Source: BMC Plant Biol. 2009 Mar 16;9:29. doi: 10.1186/1471-2229-9-29 (PMC2664812; doi:10.1186/1471-2229-9-29)
Supplement: Additional file 10 — Mean natural scale RMA values of putatively root specific genes in the 17 pistil arrays. Table of genes used to estimate expression value for genes expected to be absent in the samples used. [file 1471-2229-9-29-S10.pdf]

Additional file 10: Mean natural scale RMA values of putatively root-specific genes in the 17 pistil arrays.

Gene	Affymetrix ID	Mean value
At1g02230	264174_s_at	12.10
At1g03840	265081_at	11.79
At1g28160	259590_at	13.48
At1g47790	261734_at	9.13
At1g74080	260394_at	9.79
At2g16910	266530_at	11.34
At2g28700	263444_at	11.56
At2g32460	267087_at	19.39
At2g33720	267450_at	13.11
At3g20840	257976_at	13.06
At3g30210	257307_at	15.85
At3g49760	252232_at	9.29
At3g56560	251694_s_at	7.53
At4g18450	254674_at	13.56
At5g01860	251064_at	21.47
At5g04390	245705_at	10.08
At5g49240	248649_at	15.18
At5g52260	248343_at	9.91
At5g54230	248203_at	7.86
At5g62320	247451_at	22.55
	**Mean**	**12.90**
	**Median**	**11.79**
	**Standard deviation**	**2.16**
	**Standard error**	**0.12**
	**Maximum**	**44.83**
