# Supplementary material for: Expression-based discovery of candidate ovule development regulators through transcriptional profiling of ovule mutants
Source: BMC Plant Biol. 2009 Mar 16;9:29. doi: 10.1186/1471-2229-9-29 (PMC2664812; doi:10.1186/1471-2229-9-29)
Supplement: Additional file 11 — Genes tested with qRT-PCR and primers used. Table of genes validated with qRT-PCR. [file 1471-2229-9-29-S11.pdf]

Additional file 11: Genes tested with qRT-PCR and primers used.

Gene	Name or protein domain	Product Length	Primers	Primer sequence	primer position
AT4G27090[#]	60S RPL14	*232*	RPL14B-Fw	GAGAGGATCCAGATGAACTTCAAGA	exon-exon
			RPL14B-Rv	GCTCTTGCCTGACAACACCAGCTTT	
AT1G23420	INO	*106*	INOqFW1	GGTGAATCAGGAGAAGGAGAACAG	exon-exon
			INOqRV1	CTCAGGTGGTTTATTGACAACTTGG	
AT1G68190	Zn Finger Bbox	*78*	1g68190qFW1	TCCCTCCCTCTCTAACAATACACG	exon-exon
			1g68190qRV1	TTCTCCTCCACTCTTGCTCTCTTC	
AT1G71030	Myb domain	*134*	1g71030qFW1	ATAGATGGTCATTGATAGCGGGAAG	no intron
			1g71030qRV1	GTGATGGTGGAGACGATGATTGG	
AT1G79700*	ANT-like	*189*	1g79700qFW1	AGCGTCTGCGTCTGAGTCTG	intron incl
			1g79700qRV1	CGTCCTTTCTTGGTCTGTGTATCG	
AT2G01500	WOX6	*124*	2g01500qFW1	CGTCGTGAAGGTGGTGCTATTATC	intron incl
			2g01500qRV1	GTGGCTGTGGTCGGTTTGTG	
AT2G33830	kinase	*78*	2g33830qFW1	TGACATCAAAGGAGAAGGGAGCAG	exon-exon
			2g33830qRV1	CCTGGCGTCGTCGGAGTTC	
AT2G34700	OLE E1 allergen	*290*	2g34700qFW1	TTCAAGGAGCGACCGTAAAGC	exon-exon
			2g34700qRV1	GAAGGGACCAACAGAGAAGAGAAC	
AT3G54340	AP3	*165*	3g54340qFW1	AGCCCTAACACCACAACGAAG	exon-exon
			3g54340qRV1	CAAACACTCACCTAGCCTCTGC	
AT5G03790	HB L-zip class I	*118*	5g03790qFW1	CTTACGCAGCAGGAAATTCATATACG	exon-exon
			5g03790qRV1	ATCTCATTGTTGTTGTTCGGAAATCG	
AT5G42630	ATS/KAN4	*201*	5g42630qFW1	GGTAGAGAAAGAGGCAGAGCAGAG	intron incl
			5g42630qRV1	TTGTCCTTAGTGTGATGAGTTGTTCC	
AT5G57720	B3 domain	*125*	5g57720qFW1	CAAGAAAGGAAGGGTTACAAGAAAGC	exon-exon
			5g57720qRV1	ATATACGCCTGGAACACGATGAAG	
AT2G23060	GNAT family	*153*	2g23060qFW1	ATGCTGGTGGCTGAGATTGG	exon-exon
			2g23060qRV1	AAGTGGTTTGGTAATAACGACATCG	
AT4G36740	HB L-zip class I	*86*	4g36740qFW1	ACAACCAGTAGGAGAAGTGAAGC	exon-exon
			4g36740qRV1	AACCGTTGCCTCCATCTGC	
AT5G17300	Myb domain	*152*	5g17300qFW1	GTTATGGCGTCGTCTCCGTTG	exon-exon
			5g17300qRV1	GTGTATGGCTTCCGTACCTTGG	

*At4g27090 was used as the cellular reference standard.
